# Heme promotes transcriptional and demethylase activities of Gis1, a member of the histone demethylase JMJD2/KDM4 family

**DOI:** 10.1093/nar/gkx1051

**Published:** 2017-11-06

**Authors:** Sneha Lal, Jonathan M Comer, Purna C Konduri, Ajit Shah, Tianyuan Wang, Anthony Lewis, Grant Shoffner, Feng Guo, Li Zhang

**Affiliations:** Department of Biological Sciences, University of Texas at Dallas, Mail Stop RL11, 800 W. Campbell Road, Richardson, TX 75080, USA; Diabetes Center, University of California San Francisco, San Francisco, CA 94143, USA; Department of Biological Chemistry, David Geffen School of Medicine, University of California, Los Angeles, CA 90095, USA

## Abstract

The yeast Gis1 protein is a transcriptional regulator belonging to the JMJD2/KDM4 subfamily of demethylases that contain a JmjC domain, which are highly conserved from yeast to humans. They have important functions in histone methylation, cellular signaling and tumorigenesis. Besides serving as a cofactor in many proteins, heme is known to directly regulate the activities of proteins ranging from transcriptional regulators to potassium channels. Here, we report a novel mechanism governing heme regulation of Gis1 transcriptional and histone demethylase activities. We found that two Gis1 modules, the JmjN + JmjC domain and the zinc finger (ZnF), can bind to heme specifically *in vitro. In vivo* functional analysis showed that the ZnF, not the JmjN + JmjC domain, promotes heme activation of transcriptional activity. Likewise, measurements of the demethylase activity of purified Gis1 proteins showed that full-length Gis1 and the JmjN + JmjC domain both possess demethylase activity. However, heme potentiates the demethylase activity of full-length Gis1, but not that of the JmjN + JmjC domain, which can confer heme activation of transcriptional activity in an unrelated protein. These results demonstrate that Gis1 represents a novel class of multi-functional heme sensing and signaling proteins, and that heme binding to the ZnF stimulates Gis1 demethylase and transcriptional activities.

## INTRODUCTION

Many JmjC domain-containing proteins possess demethylase activity and can remove specific methyl groups on histones or other proteins. They are dioxygenases that use α-ketoglutarate and Fe^2+^ to oxidize various substrates (1–3). In humans, 32 JmjC domain-containing proteins have been identified (4). These proteins have fundamental biological functions, and their dysfunctions are implicated in many pathological processes, including developmental deficiency, cancer and cardiovascular diseases (4–6). Likewise, heme (iron protoporphyrin IX) plays key physiological and pathological roles in virtually all living organisms (7). As an essential prosthetic group and cofactor in many proteins and enzymes, heme is required for the proper functioning of the mitochondrial respiratory chain complexes; the synthesis and sensing of CO and NO; and the activity of many enzymes involved in the transport, storage and utilization of oxygen, such as cytochrome c and P450s (8–10). Further, heme serves as an important signaling molecule that directly regulates diverse molecular and cellular processes ranging from gene transcription and translation to microRNA processing and potassium channel activation (11–16). Recent epidemiological and experimental studies have implicated altered heme availability in the development and progression of an array of common human diseases, including cancer, diabetes and cardiovascular diseases (17,18).

**Figure 1.**
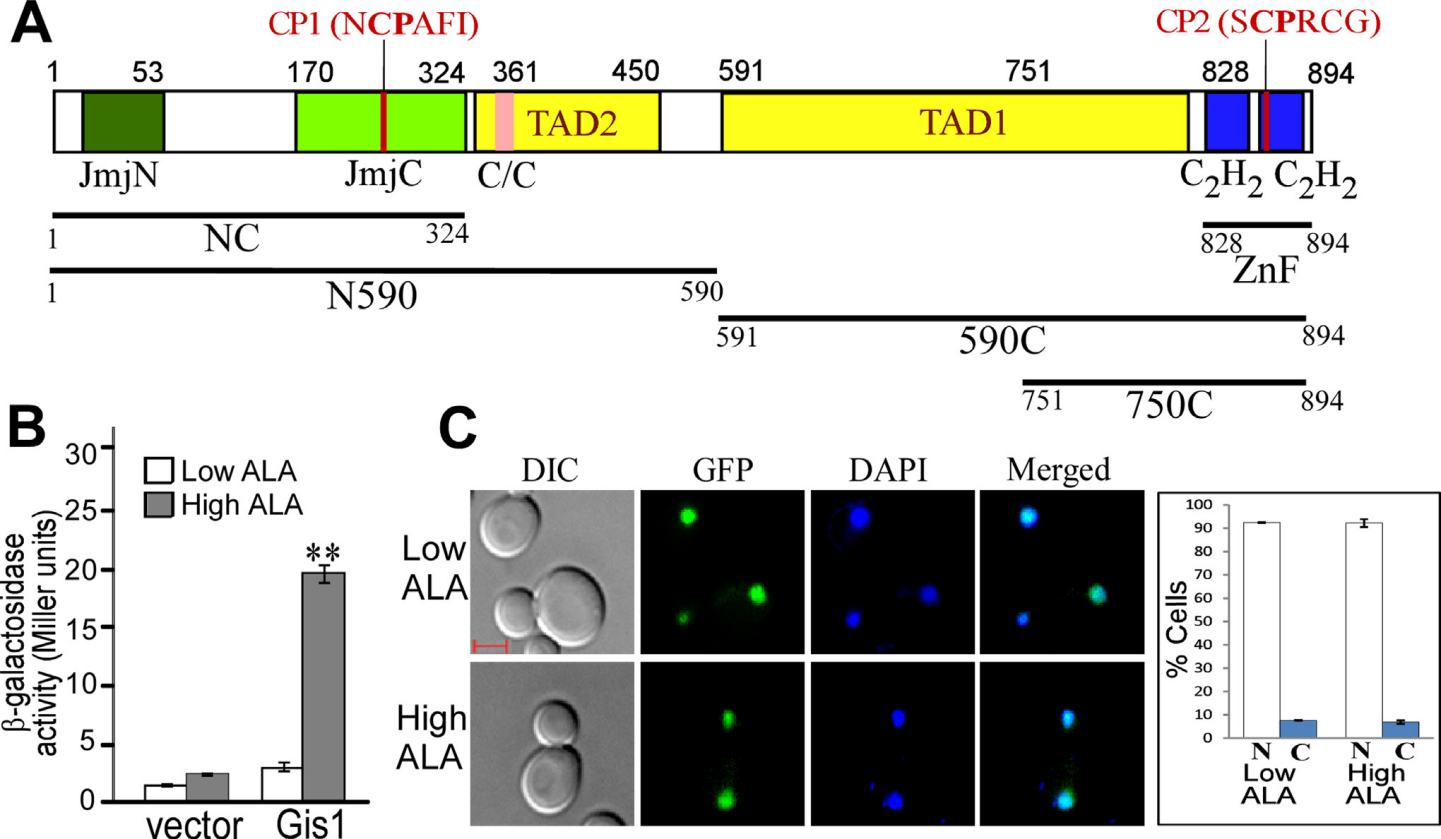
(**A**) The domain structure of Gis1 protein. Shown here are the previously identified JmjN + JmjC domain, coiled-coil domain (C/C), two C2H2 type ZnFs and twoTADs (TAD1 and TAD2). Also shown are two CP motifs. (**B**) The effect of heme on Gis1 transcriptional activity. Yeast *Δgis1Δhem1* bearing the expression plasmid for Gis1 under the control of its native promoter or the empty vector and the PDS-*lacZ* reporter were grown in the presence of a low (2.5 μg/ml) or high (250) μg/ml level of heme precursor 5-aminolevulinc acid (ALA) until post- diauxic shift to activate Gis1. Then, cells were collected and β-galactosidase activities were measured. The plotted values are averages from at least three independent cultures. Standard errors of measurements are plotted. For statistical analysis, the activities in cells grown under heme-deficient and -sufficient conditions were compared with a Welch two-sample *t*-test. **, *P*-value, 2.4 × 10^−5^. (**C**) Images showing the effect of heme on subcellular localization of Gis1. DIC, GFP, DAPI and merged fluorescent images of cells expressing Gis1-GFP, grown in the presence of a low (Low) or high (High) level of ALA. The cells showing GFP-tagged proteins in the nucleus (N) or cytosol (C) were counted, and the values are plotted here. The scale bar represents 1 μm.

Previously, the yeast protein Gis1 was shown to be a transcriptional regulator also belonging to the JMJD2/KDM4 subfamily of demethylases (19–21). The JmjN + JmjC domain in Gis1 is highly homologous to the JmjN + JmjC domains in mammalian JMJD2/KDM4 proteins, which possess demethylase activity (19) (Supplementary Figure S1). These proteins play important roles in histone methylation, oxygen regulation and hormonal signaling (3,5,6,22). Gis1 binds to the PDS (post-diauxic shift) element (23,24) and modulates the transcription of hundreds of genes involved in nutrient signaling, oxidative stress signaling and aging (25–30). Gis1 can both activate and repress transcription (24,31). Additionally, several studies have shown that Gis1 possesses weak to moderate demethylase activity toward histone H3 (20,21,32). Gis1 contains multiple domains: a JmjN region, a JmjC region, a coiled-coil domain, two C2H2 type zinc fingers (ZnFs) and two transcription activation domains (TADs) (Figure 1A) (28,33,34). JmjN and JmjC interact physically to form a structural unit or a domain (34). The JmjN + JmjC domain presumably confers histone demethylase activity, but is dispensable for transcriptional activation by Gis1 (33). Intriguingly, a survey of the protein sequence identified two Cys Pro (CP) motifs, also known as heme regulatory motifs (HRMs). One is located in the JmjC domain, and another one in the C2H2 ZnF (Figure 1A). While a wide array of peptides containing HRM or CP motifs bind to heme reversibly in the micromolar range (35,36), the existence of HRMs in proteins does not necessarily indicate heme binding by the proteins or heme responsiveness in protein activity. For example, only one or some of the CP motifs in transcriptional regulators Hap1 and Bach1 are essential for heme regulation (7,35,37–39). Structural environment dictates whether CP motifs play a role in heme regulation (40). Direct biochemical and functional studies are necessary to determine heme binding and heme regulation of interested proteins. Notably, Gis1 is oxygen sensitive, and oxygen signaling can be mediated by heme (41–43). We therefore explored the possibility that Gis1 activity is regulated by heme.

We found that Gis1 transcriptional and demethylase activities are regulated by heme, although heme is not required for Gis1 nuclear localization. Further biochemical analyses showed that at least two regions of Gis1 can bind heme *in vitro*, namely the JmjN + JmjC domain and the ZnF. The JmjN + JmjC domain is not required for heme activation of Gis1 activity, but confers histone demethylase activity. Additionally, we showed that heme does not affect Gis1 DNA binding *in vitro*. We found that the Gis1 regions mediating heme binding and regulation can confer heme regulation via an unrelated DNA-binding domain. These results demonstrated that Gis1 is a novel, multi-functional signaling protein, which senses intracellular heme levels and modulates its transcriptional and demethylase activities.

## MATERIALS AND METHODS

### Yeast strains and plasmids

The yeast strains used were BY4741Gis1-GFP*Δhem1* (*MAT*a *his3Δ1 leu2Δ0 met15Δ0 ura3Δ0 hem1-Δ100 GIS1-GFP*), MHY101 (*MAT*a *ura3–52 leu2–3,112 his4–519 ade1–100 hem1-Δ100 URA3::PDS-lacZ*) and MHY101*Δgis1* (*MAT*a *ura3–52 leu2–3,112 his4–519 ade1–100 hem1-Δ100 URA3::PDS-lacZ gis1::LEU2 ura3::Kan^r^*). To delete the *GIS1* gene, MHY101 cells were transformed with a polymerase chain reaction (PCR) product containing *LEU2* gene in the middle and 44-bp sequences flanking the open reading frame sequence of *GIS1* on both sides. Knockout strains were confirmed by PCR and β-galactosidase assay. The PDS element-driven *LEU2-lacZ* reporter was introduced by transforming yeast strains with the linearized, NcoI-cut pLS9-PDS plasmid, as described previously (24). In the MHY101*Δgis1* strain, the *URA3* gene was deleted by transformation with a PCR product containing Kan^r^ sequence in the middle and 44 bp of sequence flanking the open reading frame sequence of *URA3* on both sides. The *HEM1* gene in the BY4741Gis1-GFP strain was deleted as described previously (44).

The PDS element-driven *LEU2-lacZ* reporter plasmid pLS9-PDS is as described (24), and was provided by Dr Claudio de Virgilio’s lab (University of Fribourg, Switzerland). The pET-15b bacterial expression vector for expressing His_6_-tagged Gis1 from the T7 lac promoter is as described (45), and was provided by Dr George M. Carman’s lab (Rutgers University, New Jersey). The expression vectors for N590, NC, 590C, 750C, ZnF and Gis1ΔZnF (with the ZnF residues 828–894 deleted) were constructed by replacing the full-length Gis1 DNA in the pET-15b vector with DNA containing coding sequences for the Gis1 fragments. The coding sequences were confirmed by DNA sequencing (Eurofins MWG operon USA). The yeast expression vectors for full-length Gis1 (pYY53), Gis1ΔJmjC (pYY54) and Gis1ΔZnF (pYY55) are as described (33), and were provided by Dr Rolf Sternglanz’s lab (Stony Brook University, NY, USA). The expression vectors for Gis1ΔJmjN, Gis1ΔJmjN/C and Gis1ΔJmjN/C/CC are as described (34,46), and were provided by Dr Nianshu Zhang’s lab (University of Cambridge, UK). The expression vectors for fusion proteins HG1, HG2, HG2_mut_ and HG3 were constructed by replacing the Hap1-coding sequences in the yeast expression vector SD5-HAP1 (47) with corresponding Gis1-coding sequences. The DNA containing the coding sequences for fusion proteins was generated by overlapping PCR, as described previously (48). Other inactive fusion proteins containing the Hap1 DNA-binding domain and various Gis1 regions were constructed in the same way. Gis1 mutants with point mutations were generated by using the QuikChange II XL Site-Directed Mutagenesis Kit (Agilent Technology). The sequences of mutant and fusion proteins were confirmed by DNA sequencing by Eurofins MWG operon USA (Louisville, KY, USA).

### Cell growth, β-galactosidase assays and immunofluorescence staining

Yeast cells were grown in rich YPD or synthetic complete media, as described previously (49,50). Cell density was determined by measuring optical density at 600 nm. To determine β-galactosidase levels from reporter genes in *Δhem1* cells bearing the PDS element-driven *LEU2-lacZ* reporter or the Hap1-driven UAS1-*CYC1-lacZ* reporter, cells were grown in synthetic complete medium containing a limiting amount of the heme precursor 5-aminolevulinic acid (ALA) 2.5 μg/ml or a high amount of ALA (250 μg/ml). Cells were collected after diauxic shift for measuring Gis1 activity or after they reached A_1.0–1.5_ for measuring Hap1 activity. Collected cells were then subjected to chloroform permeabilization and β-galactosidase assays. The activities were measured and calculated in Miller units, as described previously (51,52). For immunofluorescence staining, cells bearing the expression vector for FLAG-tagged Gis1 or deletion proteins were grown in synthetic complete media to A_0.8–1.0_. Subsequent fixing and staining of cells were carried out as described (53,54). Briefly, cells were fixed with 5% formaldehyde for 90 min and permeabilized with Zymolyase. Cells were incubated with an anti-FLAG antibody overnight at room temperature, and then incubated with an affinity-purified, fluorescein-conjugated anti-rabbit IgG goat antibody for 2 h. Finally, cells were stained with DAPI for 10 min before viewing and imaging.

### Purification of Gis1 proteins, spectroscopic analyses, protein binding to heme-agarose beads and protease sensitivity assay

To purify full-length Gis1 and various Gis1 fragments from *Escherichia coli*, BL21(DE3) bearing the pET-15b expression plasmids (45) were grown to A_0.5_, then induced with 1 mM Isopropyl β-D-1-thiogalactopyranoside for 2 h at 25°C. Cells were collected and lysed with a French Press. The His_6_ tagged Gis1 proteins were purified with Ni Sepharose 6 fast flow columns (GE Healthcare) according to the manufacturer's protocol. The eluted His_6_-Gis1 proteins were desalted with the PD-10 desalting columns (GE healthcare), then cleaved with 0.005U thrombin per μg of protein. Gis1 proteins without the tag were then purified from the mixture by passing through Ni Sepharose 6 fast flow columns. To overcome nonspecific Ni binding, a stepped elution was performed with elution buffer containing increasing concentrations of imidazole (0, 40, 80 and 250 mM). All the eluates were analyzed on sodium dodecylsulphate-polyacrylamide gel electrophoresis (SDS-PAGE) gels.

Heme absorbance spectra were measured by using a Varian Cary^®^ 50 UV-Vis Spectrophotometer. Samples contained 10 μM protein, 5 μM Heme, and 0, 5, 10, 20, 40, 80, 160 or 250 mM imidazole. Protein and heme were prepared in 20 mM Tris, pH 8.0 and 500 mM NaCl; the imidazole stock was adjusted to pH 8.0 with HCl. Each sample was incubated for 30 s after the addition of heme, prior to absorbance measurement.

To detect Gis1 protein binding to heme by using heme agarose beads (Sigma), purified proteins were incubated with the beads in the heme binding buffer (20 mM Tris pH 8.0, 500 mM NaCl and 1% Triton X-100) for 1 h at 4°C while rotating at 200 rpm. After incubation, the beads were pelleted by centrifugation, and the supernatant was collected. The beads were then washed twice with heme binding buffer. Subsequently, proteins bound to the beads and in the supernatant were electrophoresed on SDS-PAGE and visualized by Coomassie blue staining.

For elastase sensitivity assay, 20 μM purified proteins were pre-incubated with heme (35 μg/ml) for 10 min in 20 mM Tris, pH8.0, 500 mM NaCl. Then, elastase was added to the proteins for 10 min. The reactions were stopped by adding SDS loading buffer, and samples were analyzed by SDS-PAGE.

### Size exclusion chromatography

The SEC experiments were performed at room temperature using an AKTA Purifier chromatography system and a Superdex 200 10/300 GL column. The 1× phosphate buffered saline (PBS) running buffer included 10.1 mM Na_2_HPO4, 1.76 mM KH_2_PO4, 137 mM NaCl and 2.68 mM KCl at pH 7.6. The NC and 750C proteins were mixed with hemin in 1× PBS at concentrations specified in Figure 5 and incubated at room temperature for 30 min before injecting to the column.

### Measurement of Gis1 demethylase activity

The demethylase activity of purified Gis1 proteins was measured with a custom-made Epigenase Demethylase Activity/Inhibition Assay Kit (EpiGentek, P3081–48 CUSTOM) according to the manufacturer's protocol. Briefly, 50 ng of the di-methyl histone H3K36 peptide, which was derived from residues within 1–100 of human histone H3 (Cat. No. R-1038), was stably coated onto 96-well microplate wells. Enzymatic reactions were set up for blank (without protein) and sample (with 300 ng or about 3 pmol of purified Gis1 proteins) in a total volume of 50 μl, in the presence or absence of 2 μM heme. The microplate strip wells were covered with adhesive covering film and incubated at 37°C for 120 min. To detect the amount of demethylated product, the wells were washed and incubated with a capture antibody (anti-H3K36me1) at room temperature for 60 min followed by incubation with a detection antibody at room temperature for 30 min. The amount of demethylated products was fluorometrically measured with a BioTek Cytation 5 plate reader with excitation and emission wavelengths of 530 and 590 nm, respectively, after adding a fluorescence development solution. Demethylase activity of the protein was calculated in RFU/min/mg as described in the manufacturer’s protocol.

### Preparation of yeast extracts, western blotting, electrophoretic mobility shift assays and ChIP-qPCR

For preparation of yeast cell extracts, *Δgis1* cells bearing the expression vector for full-length Gis1 or Gis1 deletion proteins were grown in synthetic complete media to A_1.0–1.5_. Cells were harvested and extracts were prepared as described previously (55). Protein concentrations were determined with the BCA assay kit (Thermo Scientific). For Western blotting, 75 μg of proteins from each treatment condition were electrophoresed on 10% SDS-PAGE, then transferred onto the Immuno-Blot PVDF Membrane (Bio-Rad). The membranes were probed with antibodies, followed by detection with a chemiluminescent western blotting kit (Roche Diagnostics). The signals were detected with a Carestream image station 4000MM Pro, and quantitation was performed with the Carestream molecular imaging software version 5.0.5.30 (Carestream Health, Inc.).

For electrophoretic mobility shift assays (EMSA), DNA-binding reactions were carried out in a 20 μl volume with 5% glycerol, 4 mM Tris (pH 8.0), 40 mM NaCl, 4 mM MgCl_2_, 10 mM dithiothreitol (DTT), 3 μg of salmon sperm DNA and 300 μg of bovine serum albumin per ml in the presence or absence of 2 μM heme. Approximately 0.01 pmol of labeled UAS1/*CYC1* or PDS and 20 μg of protein extracts were used in each reaction. The reaction mixtures were incubated at room temperature for 1 h and then loaded onto 4% polyacrylamide gels in one-third × Tris-borate-ethylenediaminetetraacetic acid for PAGE at 4°C. For antibody super shifts of the DNA–protein complexes, anti-Hap1 or anti-FLAG antibodies or pre-immune serum was added to the reaction and were incubated for 40 min, followed by electrophoresis on 4% nondenaturing polyacrylamide gels.

Chromatin immunoprecipitation (ChIP) was carried out as described previously (52). Briefly, yeast MHY101*Δgis1* cells bearing the expression vector for full-length Gis1-FLAG or an empty vector were grown to A_1.2_ under heme-deficient or heme-sufficient conditions. Cells were collected and fixed with formaldehyde for 30 min at room temperature and then resuspended in lysis buffer. Cells were permeabilized by agitation with equal volumes of glass beads. Permeabilized cells were sheared using a sonicator. Cell extracts were collected and incubated with magnetic beads (Dynabeads Protein G, Novex Thermo Fisher) bound with or without anti-FLAG antibodies (Sigma). Bound complexes were eluted using elution buffer. Subsequently, crosslinking was reversed by incubating with 1% SDS at 65°C overnight. DNA was collected and subjected to qPCR analysis (LightCycler FastStart DNA MasterPlus SYBR Green I, Roche) using primers for GRE1 promoter (FW 5′ GGTTCCAGGTATGGGTTTGA, RV 5′ ACAACTAAGGCAAAACTGCC). Percentage of input was calculated using the ΔCt formula: (2^−(Ct IP—Ct Input X DF)^)× 100.

## RESULTS

### The activation of Gis1 transcriptional activity requires heme

We examined whether heme modulates Gis1 transcriptional activity using a PDS (post-diauxic shift) element-driven *lacZ* reporter (24). Gis1 binds to PDS and activates genes induced by nutrient limitation following diauxic shift, such as *GRE1* (24,45,46). We measured the reporter activity in heme-deficient and heme-sufficient cells following diauxic shift. We found that a high level of intracellular heme was required for the activation of Gis1 transcriptional activity (Figure 1B). Additionally, by imaging live cells expressing Gis1-GFP from the chromosomal location of the *GIS1* gene (56), we found that Gis1 localizes entirely to the nucleus even in heme-deficient cells (Figure 1C).

### Gis1 contains two regions that can bind to heme specifically *in vitro*

To pinpoint the Gis1 region that binds to heme *in vitro*, we purified Gis1 proteins encompassing different regions (Figure 1A): full-length Gis1, N590 (residues 1–590), NC (the JmjN + JmjC (residues 1–324), 590C (residues 591–894), 750C (residues 751–894) and ZnF (residues 828–894). Little degradation was observed for NC, 750C and ZnF (Supplementary Figure S2), whereas full-length Gis1, N590 and 590C exhibit significant degradation (Supplementary Figure S2), likely because they contain more than one structural domain and do not fold into one compact protein entity.

**Figure 2.**
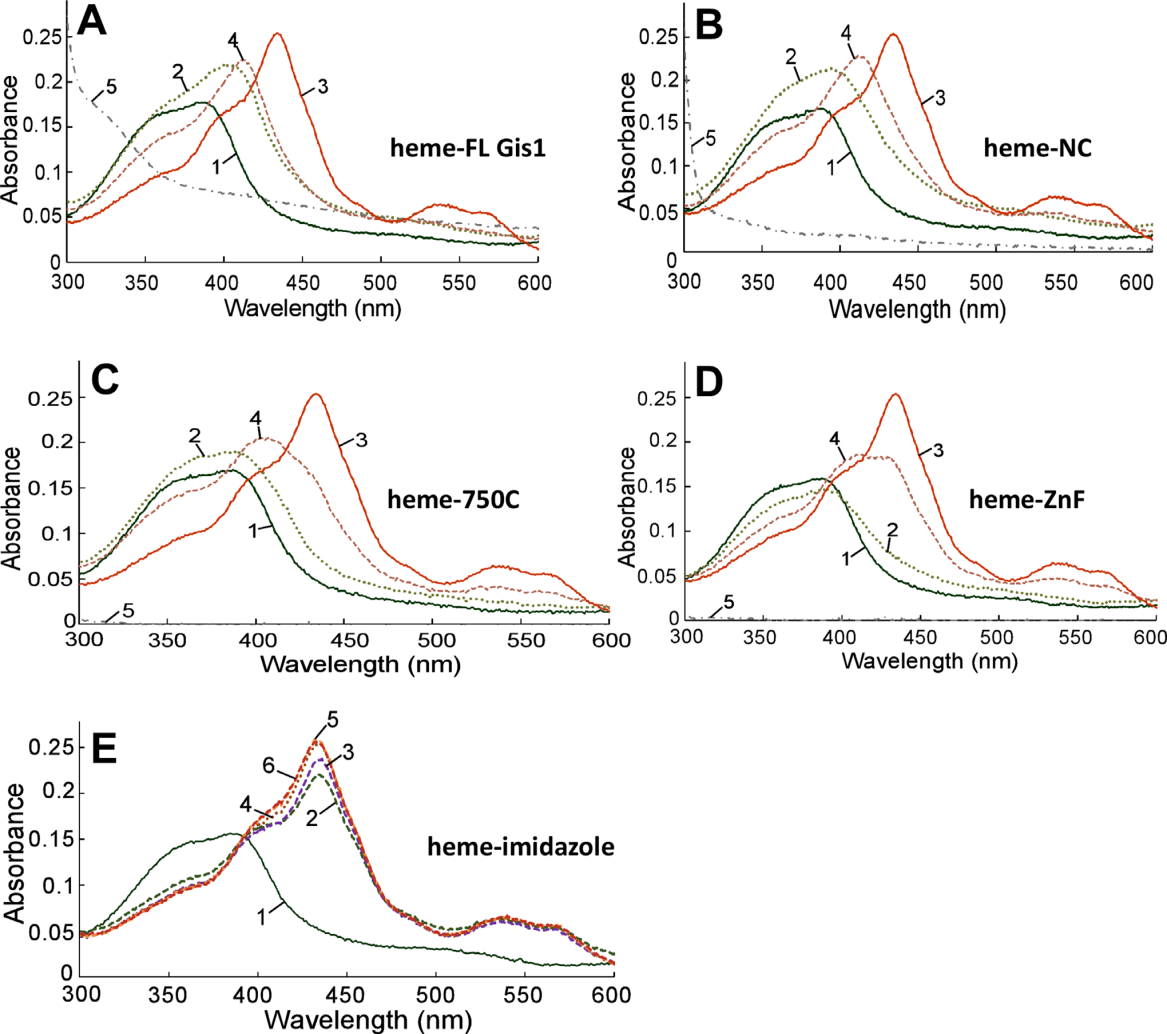
Absorption spectra of heme in the absence and presence Gis1 proteins. (**A**) Heme absorption spectra in the presence of full-length (FL) Gis1. (**B**) Heme absorption spectra in the presence of NC. (**C**) Heme absorption spectra in the presence of 750C. (**D**) Heme absorption spectra in the presence of ZnF. (**E**) The effect of increasing concentrations of imidazole on the heme absorption spectrum. In A–D, line 1: 5 μM heme, line 2: 5 μM heme + 10 μM Gis1 protein, line 3: 5 μM heme + 10 mM imidazole, line 4: 5 μM heme + 10 μM Gis1 protein + 10 mM imidazole, line 5: 10 μM Gis1 protein. In (E), line 1: 5 μM heme, line 2: 5 μM heme + 4 mM imidazole, line 3: 5 μM heme + 5 mM imidazole, line 4: 5 μM heme + 10 mM imidazole, line 5: 5 μM heme + 40 mM imidazole, line 6: 5 μM heme + 80 mM imidazole.

Firstly, we used a previously well-established method to characterize reversible heme binding to these proteins (35,57). It is worth noting that heme-protein interactions mediating signaling or regulation is often dynamic, reversible and transient. Thus, biochemical techniques designed to examine stable heme-protein binding may not detect regulatory heme–protein interactions (7,57). However, the heme spectral method can detect dynamic and transient interactions (35,57). It involves detecting the effect of peptides or proteins on the major heme absorption band, the Soret band, which is around 400 nm. All six purified Gis1 proteins bound to heme and shifted the Soret peak to longer wave lengths (Figure 2A–D). Compare lines 1 and 2 for FL (Figure 2A), NC (Figure 2B), 750C (Figure 2C) and ZnF (Figure 2D). As shown previously (35), imidazole chelates heme and shifts the Soret peak from about 386 nm to about 434 nm, and it can also enhance the spectral changes caused by protein binding (compare lines 3 and 4 in Figure 2A–D; for labile fragments N590 and 590C, see Supplementary Figure S3). Figure 2E shows that 4–80 mM imidazole induced very similar heme spectral changes.

**Figure 3.**
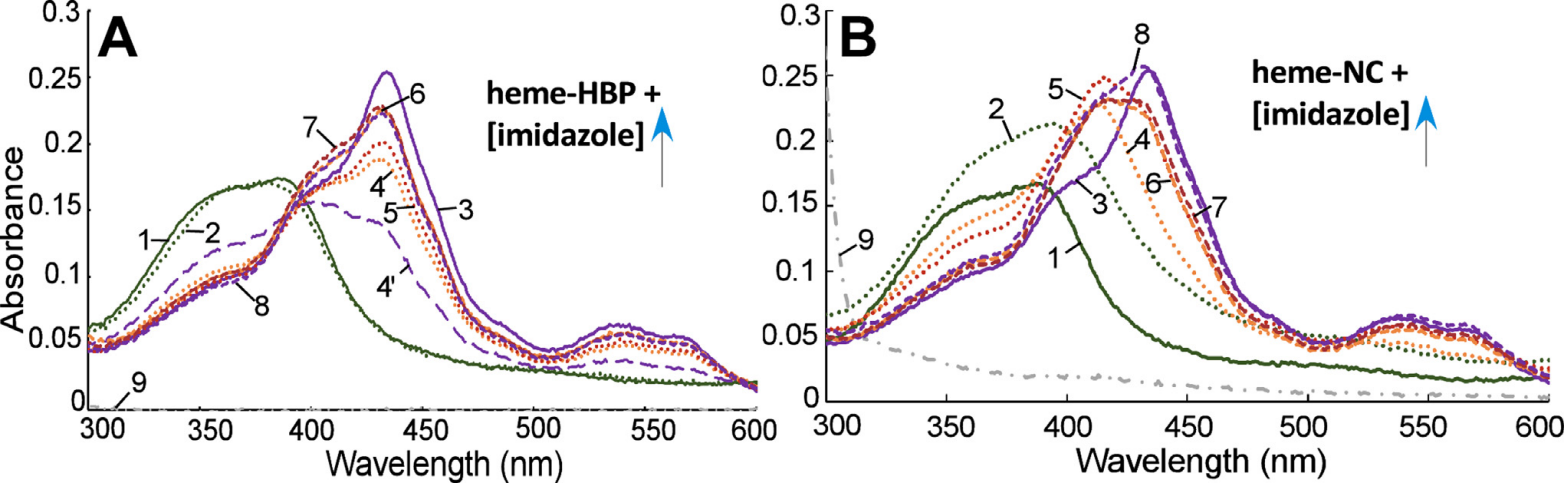
Examples of absorption spectra of heme bound to HBP or Gis1 proteins in the presence of increasing concentrations of imidazole. (**A**) The shifting of heme-HBP absorption peak from about 400 to 434 nm by increasing concentrations of imidazole. (**B**) The shifting of heme-NC absorption peak from about 406 to 434 nm by increasing concentrations of imidazole. line 1: 5 μM heme, line 2: 5 μM heme + 10 μM HBP or NC, line 3: 5 μM heme + 10 mM imidazole, line 4’ (in A): 5 μM heme + 10 μM HBP + 5 mM imidazole, line 4: 5 μM heme + 10 μM HBP or NC + 10 mM imidazole, line 5: 5 μM heme + 10 μM HBP or NC + 20 mM imidazole, line 6: 5 μM heme + 10 μM HBP or NC + 40 mM imidazole, line 7: 5 μM heme + 10 μM HBP or NC + 80 mM imidazole, line 8: 5 μM heme + 10 μM HBP or NC + 160 mM imidazole, line 9: 10 μM HBP or NC.

To estimate heme-binding affinity of Gis1 domains, we employed imidazole competition assay using a previously characterized synthetic peptide containing a CP motif (HBP: IGVVCPFVR) for reference. The HBP binds to heme with a *K*_d_ of 0.87 ± 0.41 μM (36). While the CP motif or ‘HRM’ in several proteins is essential for heme regulation, this motif *per se* is not sufficient (38,40,58). Likewise, many known CP motifs do not mediate heme regulation, and other residues, including His, Met and Tyr, can bind to heme and may mediate heme regulation. Nonetheless, the HBP provides a good reference for assessing heme-binding affinity of proteins that bind to heme reversibly and dynamically. Figure 3A illustrates heme spectral changes caused by HBP binding. The heme spectrum changed very little with the addition of HBP (compare line 1 with line 2, Figure 3A), but the presence of 5 mM imidazole enhanced the spectral changes induced by HBP binding (compare line 4’ with line 3, Figure 3A). HBP shifted the heme + imidazole peak from 434 nm to about 400 nm, reflecting heme binding mainly to HBP, instead of imidazole. However, at 10 mM imidazole, the peak was shifted back to 434 nm, reflecting heme binding mainly to imidazole. Using imidazole titration, we found that 160 mM imidazole was needed to shift the heme-NC peak to near 434 nm (Figure 3B), while 40 and 20 mM imidazole were required to shift the 750C-heme and ZnF-heme peaks to 434 nm, respectively (Supplementary Figure S4). Based on these data, we estimate that the heme-binding affinity of NC, 750C and ZnF would be approximately 20-, 8- and 4-fold higher than that of HBP. Note that the labile proteins, including FL, N590 and 590C, appear to have lower heme-binding affinity than their shorter counterparts (Supplementary Figures S3 and 4), likely because the degradation products do not bind to heme.

**Figure 4.**
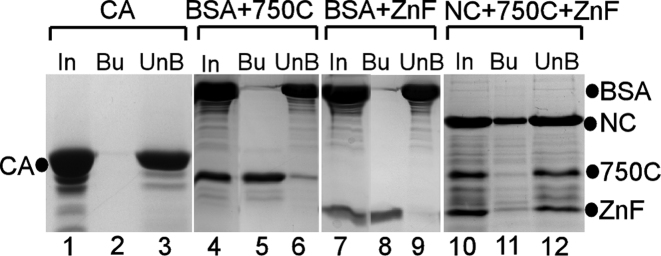
The pull-down of Gis1 proteins by heme agarose beads. Five hundred picomoles of carbonic anhydrase (CA, lanes 1–3), or a mixture of the indicated proteins (500 pmol each, lanes 4–12) were incubated with heme-agarose beads. The input (In) proteins (lanes 1, 4, 7 and 10), bound (Bu) and unbound proteins (UnB) were analyzed by SDS-PAGE and shown.

We verified the binding of Gis1 domains to heme and compared their affinity with heme agarose beads (Figure 4). Carbonic anhydrase did not bind to heme agarose (lanes 1–3, Figure 4), as expected (16). Serum albumin is well known to bind to heme directly (16,59). When 750C or ZnF were mixed in equal molar amounts with albumin (BSA) and were incubated with heme beads, they bound more strongly than BSA (lanes 4–9, Figure 4). When NC, 750C and ZnF were mixed in equal molar amounts, NC bound to heme beads much more strongly than 750C and ZnF (lanes 10–12, Figure 4).

**Figure 5.**
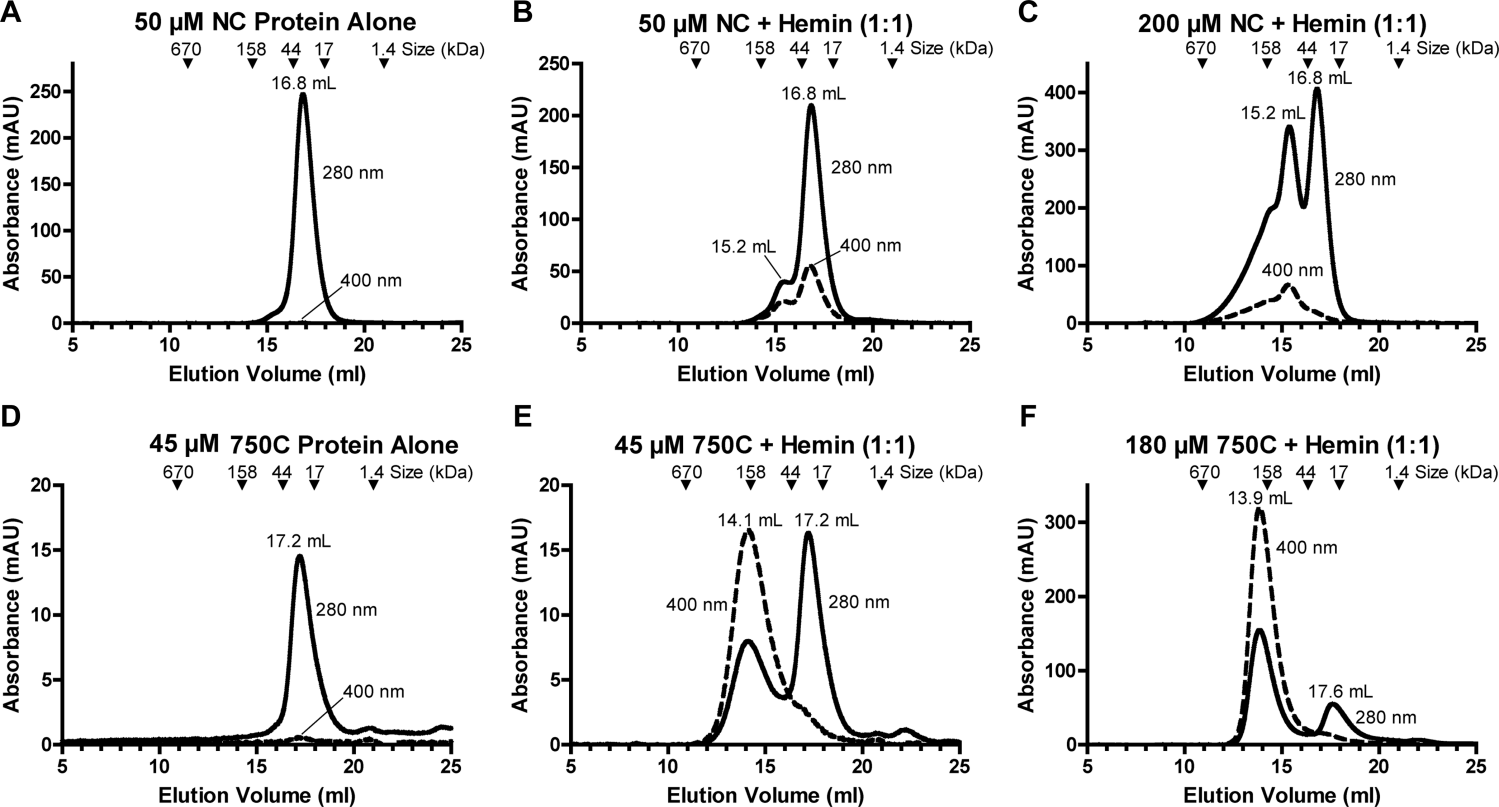
SEC analyses of Gis1-heme complexes. The NC and 750C proteins were injected with or without heme at the indicated concentrations. A Superdex 200 column and 1× PBS buffer were used in these experiments. Elution volumes of standard proteins with known molecular masses are marked.

Further, we verified heme binding to NC and 750C using size exclusion chromatography (SEC). In the absence of heme, the NC and 750C proteins eluted at 16.8 and 17.2 ml, respectively (Figure 5A and D). By comparing these elution volumes with those of standard proteins, we estimated both to be monomeric. Injection of 50 μM NC-heme complexes at 1:1 molar ratio resulted in a single major protein peak at the same elusion volume, accompanied by substantial absorbance at 400 nm, indicative of association with heme (Figure 5B). There is a minor peak with both 280 and 400 nm absorbance at 15.2 ml, which would be consistent with a heme-induced NC dimer. When the injection concentration was increased to 200 μM, the dimer peak greatly increased (Figure 5C). Under the latter condition, the monomeric peak does not exhibit strong absorbance at 400 nm, suggesting that the dimeric form has higher affinity for heme. Injection of 45 μM 750C and heme at 1:1 molar ratio resulted in a major 750C-only peak and a less intense complex peak with an elution volume of 14.1 ml (Figure 5E), which is likely to be an octamer. Doubling the amount of heme did not yield more complexes (data not shown). In contrast, increasing the concentration of the 1:1 complex injected to180 μM caused the elution profile to be dominated by the 750C–heme complex peak (Figure 5F). We conclude that heme binds to both NC and 750C proteins and promotes their oligomerization.

**Figure 6.**
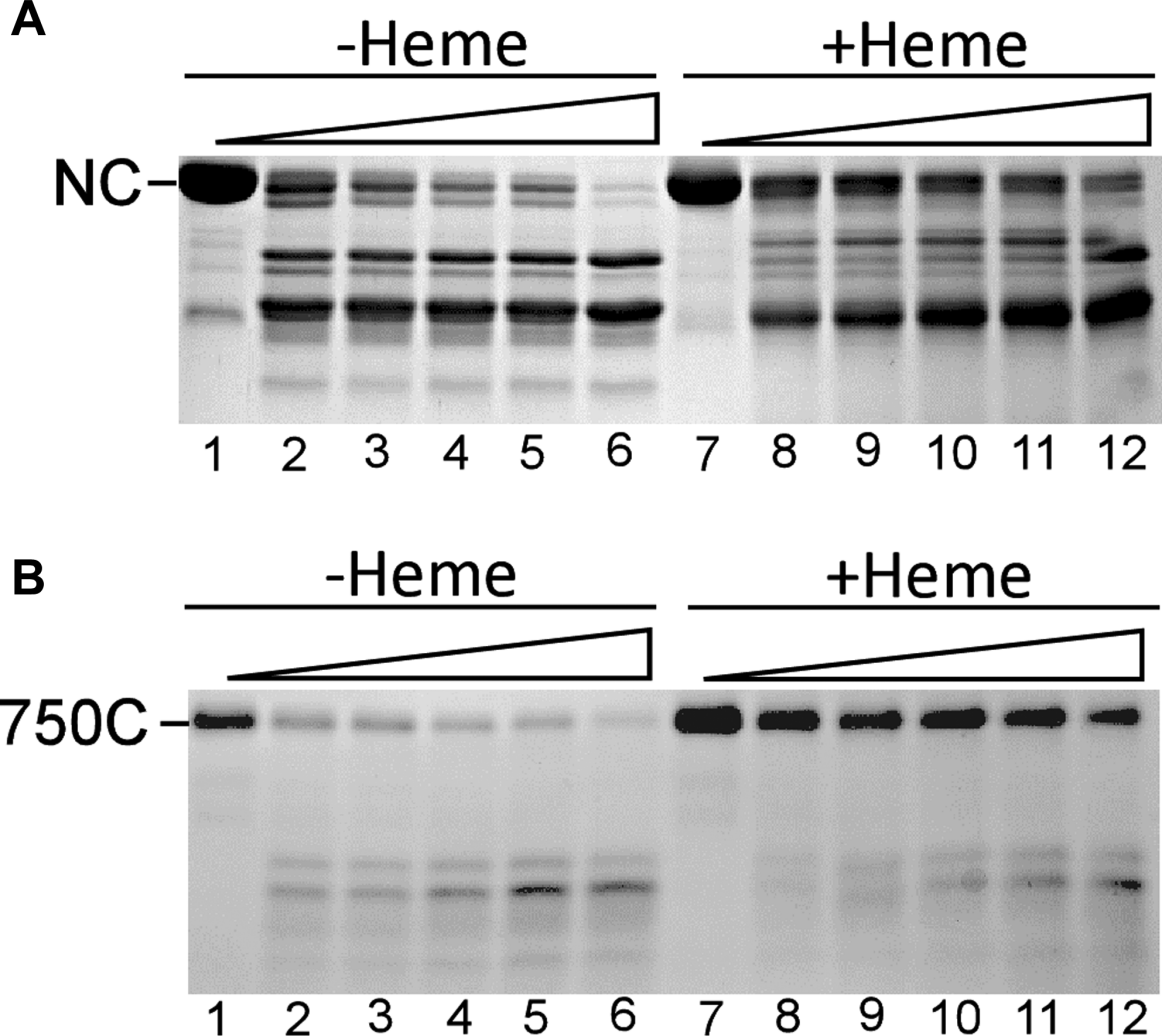
Heme binding to Gis1 domains decreases protein sensitivity to elastase. Purified NC (**A**) and 750C (**B**) protein fragments were incubated with heme, then elastase was added to the proteins with increasing concentrations. Untreated (lanes 1 and 7) and elastase-treated proteins (lanes 2–6 and 8–12) were analyzed by SDS-PAGE. Elastase concentrations in A: lanes 2 and 8, 80 ng/ml; lanes 3 and 9, 90 ng/ml; lanes 4 and 10, 100 ng/ml; lanes 5 and 11, 110 ng/ml; lanes 6 and 12, 120 ng/ml. Elastase concentrations in B: lanes 2 and 8, 2.5 ng/ml; lanes 3 and 9, 3.2 ng/ml; lanes 4 and 10, 3.8 ng/ml; lanes 5 and 11, 4.4 ng/ml; lanes 6 and 12, 5 ng/ml.

We further probed whether heme binding to NC and 750C causes protein conformational changes, which presumably indicate the potential for changes in protein function (16,60). Indeed, heme binding to NC and 750C render them substantially more resistant to elastase digestion (Figure 6A and B). In contrast, addition of heme to the non-heme-binding protein β-amylase generally did not alter its elastase sensitivity (Supplementary Figure S5). Together, the results of these spectral, chromatographic and protease sensitivity studies unequivocally demonstrate that two Gis1 modules—NC containing JmjN + JmjC and 750C containing two ZnFs—bind to heme specifically and alter their conformations.

Transcriptional activators are modular proteins with at least two separate domains, a DNA-binding domain and a TAD (e.g. see Figure 1A). TADs mediate interaction with transcriptional machinery necessary for activation, generally independently of DNA binding (61). As such, heme may activate Gis1 by stimulating DNA binding and/or by stimulating TAD activity. To determine whether heme affects DNA binding by Gis1, we performed EMSAs using purified Gis1 from *E. coli* and Gis1 in yeast extracts prepared from cells expressing Gis1 with a FLAG. Notably, addition of anti-FLAG antibody super-shifted the Gis1-DNA band, showing that the detected band represented Gis1 protein binding. For both purified Gis1 and yeast Gis1, heme did not affect DNA binding (see lanes 1–5, Supplementary Figure S6A and lanes 2–6, Supplementary Figure S6B). To ascertain the effect of heme on Gis1 DNA binding *in vivo*, we carried out ChIP coupled with quantitative PCR (ChIP-qPCR). Data in Supplementary Figure S6C suggests that heme only slightly stimulated (<2-fold) Gis1 DNA binding to DNA *in vivo*. These results together suggest that enhanced DNA binding does not contribute significantly to heme activation of Gis1 transcriptional activity.

### The ZnF, not the JmjN + JmjC domain, is essential for heme activation of Gis1 transcriptional activity

**Figure 7.**
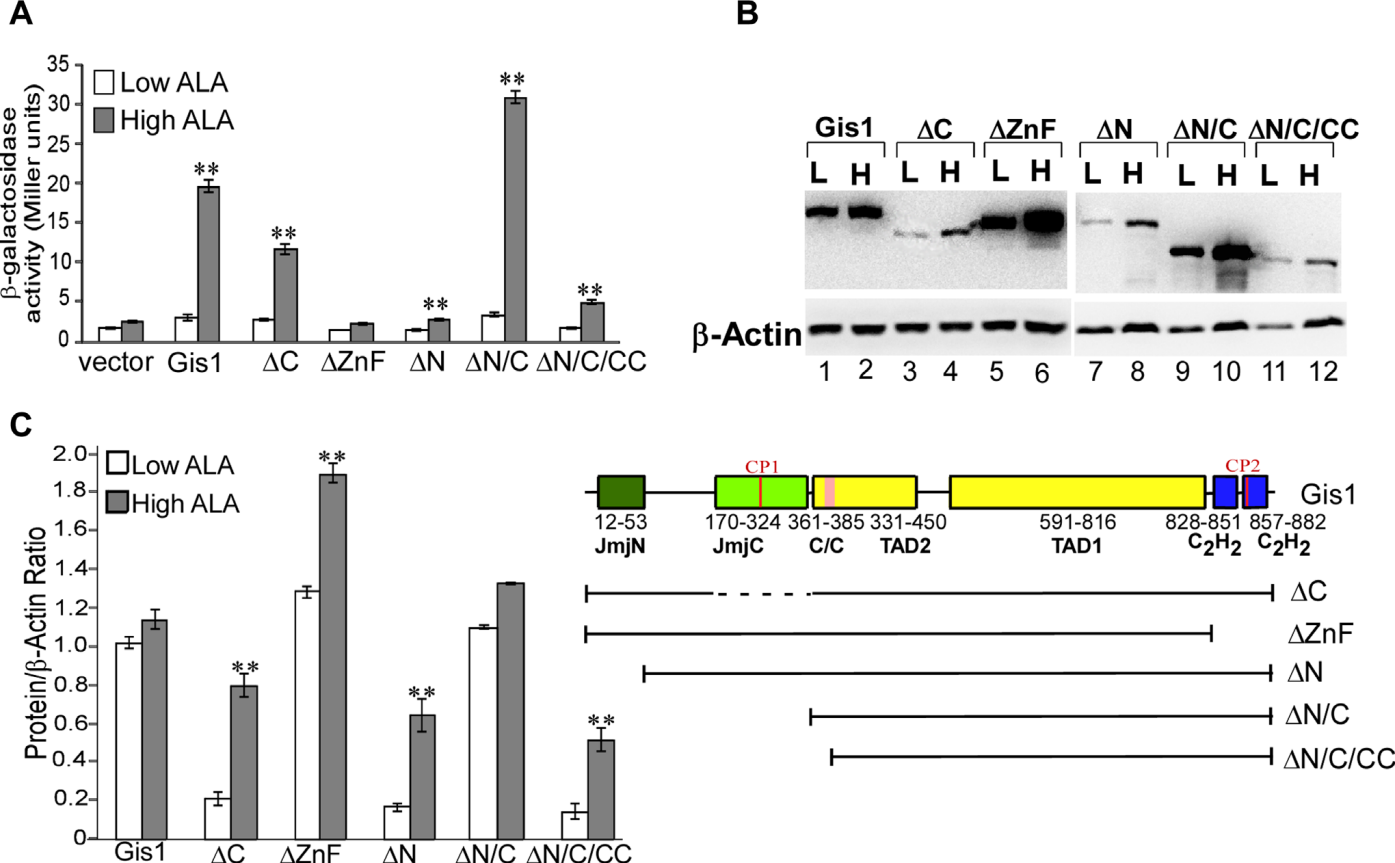
The transcriptional activities and protein levels of various Gis1 deletion proteins under heme-deficient and heme-sufficient conditions. (**A**) The Gis1 JmjN + JmjC domain is dispensable for heme activation of Gis1 transcriptional activity. Yeast *Δgis1Δhem1* cells bearing the empty vector or the expression vector for full-length Gis1, deletion mutant ΔN (JmjN deleted, see Figure 1A for Gis1 domains), ΔC (JmjC deleted), ΔN/C (JmjN + JmjC deleted), ΔN/C/CC (JmjN + JmjC and C/C deleted) or ΔZnF (ZnF deleted) under the control of its native promoter, and the PDS-*lacZ* reporter were grown in the presence of a low (2.5 μg/ml) or high (250 μg/ml) level of ALA until post-diauxic shift to activate Gis1. Then, cells were collected and β-galactosidase activities were measured. The plotted values are averages from at least three independent cultures. (**B** and **C**) Gis1 protein levels in heme-deficient and heme-sufficient cells. Yeast cells were grown as described in (A), and cell extracts were prepared and subjected to Western blotting (B). The protein levels were quantified and plotted in (C). For statistical analysis, the levels in heme-deficient cells were compared to the levels in heme-sufficient cells with a Welch two-sample *t*-test. **, *P*-value < 0.005.

To determine the functional importance of the two heme-binding regions, we detected the activities of different Gis1 deletion mutants and full-length Gis1 (for control and comparison) in heme-deficient and heme-sufficient cells. ΔN/C (with the JmjN + JmjC domain deleted) exhibited somewhat enhanced stimulation by heme when compared to full-length Gis1, while ΔC (with the JmjC region deleted) retained heme activation to a lesser extent (see Figure 7A). A previous study showed that Gis1 is negatively regulated by proteolysis (46). This is consistent with our data shown in Figure 7: all of these deletion proteins with lower protein levels, except for ΔZnF, (Figure 7C: ΔN, ΔC and ΔN/C/CC) exhibited lower transcriptional activities in heme-sufficient cells (Figure 7A). However, the slight differences in the protein levels of full length Gis1 and ΔN/C between heme-sufficient and heme-deficient conditions cannot account for much greater differences in transcriptional activities (compare Figure 7A with C). Also, immunofluorescence staining showed that full-length Gis1, ΔN/C and ΔZnF were nuclear (Supplementary Figure S7). These results show that heme activation of Gis1 transcriptional activity is not fully attributable to increased Gis1 protein levels and that the JmjN + JmjC domain is dispensable for heme activation.

**Figure 8.**
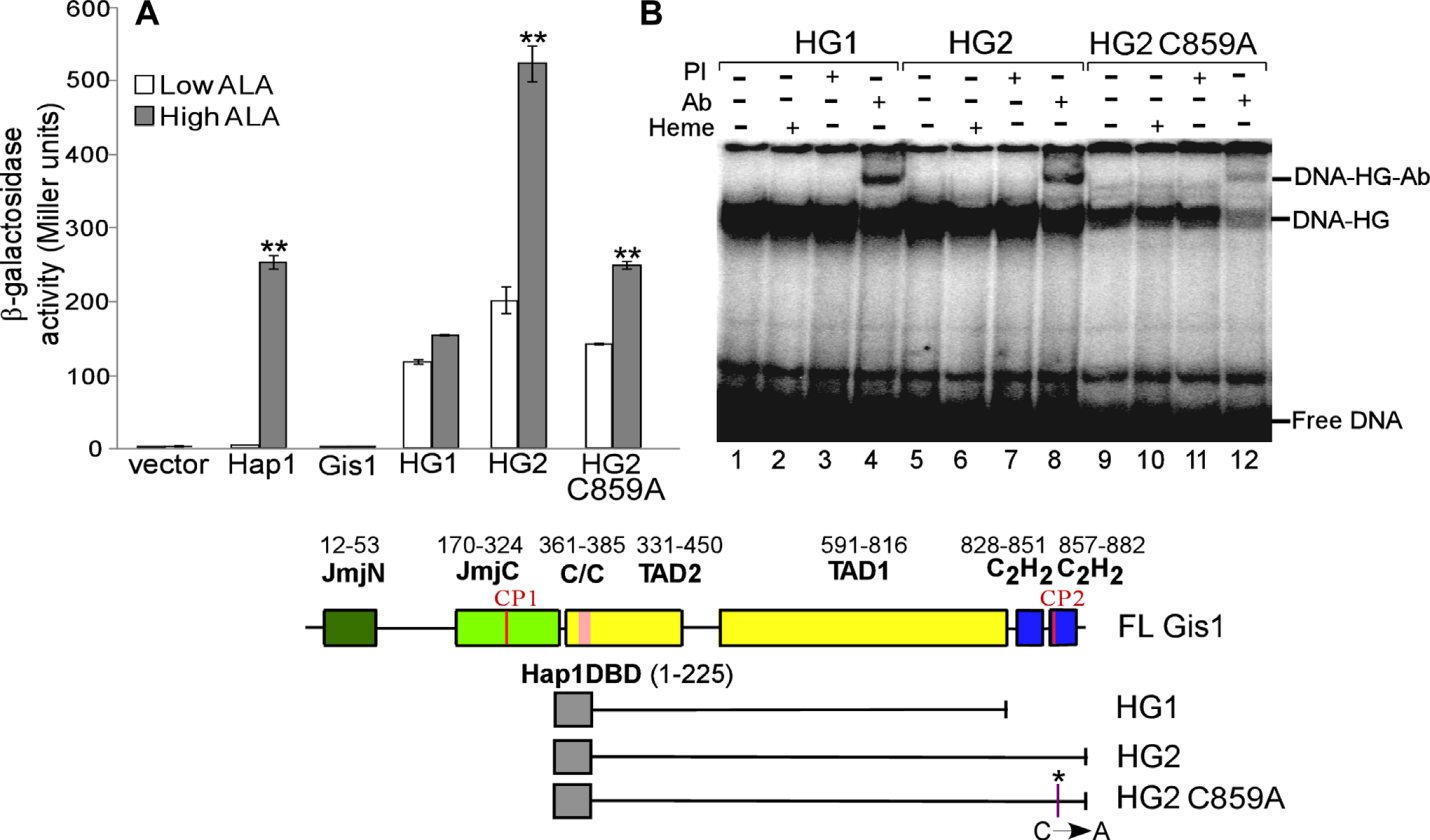
(**A**) The ZnF is essential for heme activation of Gis1 transcription-activating activity. Yeast *Δgis1Δhem1* cells bearing the empty vector or the expression vector for Hap1, Gis1, HG1 (containing Hap1 DNA-binding domain residues 1–225 and Gis1 residues 386–828), HG2 (containing Hap1 residues 1–225 and Gis1 residues 386–894) or HG2 C859A (HG2 with the Cys residue in the CP2 motif changed to Ala), and the Hap1-binding UAS1-*CYC1-lacZ* reporter were grown in the presence of a low (2.5 μg/ml) or high (250 μg/ml) level of ALA. Then, cells were collected and β-galactosidase activities were measured. For statistical analysis, the levels in heme-deficient cells were compared to the levels in heme-sufficient cells with a Welch two-sample *t*-test. **, *P*-value < 0.005. (**B**) Heme does not affect DNA binding by fusion proteins. EMSAs were performed with extracts prepared from cells expressing HG1 (lanes 1–4), HG2 (lanes 5–8) and HG2 C859A (lanes 9–12), respectively, as described in ‘Materials and Methods’ section. Anti-Hap1 antibodies were added in lanes 4 and 8, while pre-immune serum (PI) was included in lanes 3 and 7.

To define Gis1 residues mediating heme activation of Gis1 transcriptional activity, we engineered two fusion proteins: HG1, containing Gis1 residues 386–828 (lacking the ZnF; see Figure 1A for Gis1 domains) and an unrelated DNA-binding domain (Hap1 residues 1–225, no CP motif present); and HG2, containing Gis1 residues 386–894 (with the ZnF) and the Hap1 DNA-binding domain. Figure 8A shows that both fusion proteins exhibited high transcriptional activity at the Hap1-driven UAS1-*CYC1-lacZ* reporter in heme-deficient cells. Interestingly, heme further activated the activity of HG2, but not HG1. Note that other fusion proteins, including those containing full-length Gis1 or Gis1 lacking the ZnF, were labile and inactive *in vivo*, likely because altered domain architecture disrupts protein function. Further, when CP2 was mutated (Cys859 to Ala, HG2C859A, Figure 8A), the degree of heme activation was reduced. The data clearly show that ZnF is essential for heme activation of Gis1 transcriptional activity. Additionally, by performing EMSAs with yeast extracts prepared from cells expressing fusion proteins, we found that HG1, HG2 and HG2C859A all bind to the Hap1-binding site strongly, independently of heme (Figure 8B), as expected. We confirmed that the bands contain the fusion proteins by super shifting with anti-Hap1 antibodies (see lanes 4, 8 and 12, Figure 8B). Together, the results show that the Gis1 ZnF promotes heme activation of transcriptional activity, independently of DNA binding.

### The Gis1 JmjN + JmjC domain can mediate heme regulation when fused to an unrelated transcriptional activator

**Figure 9.**
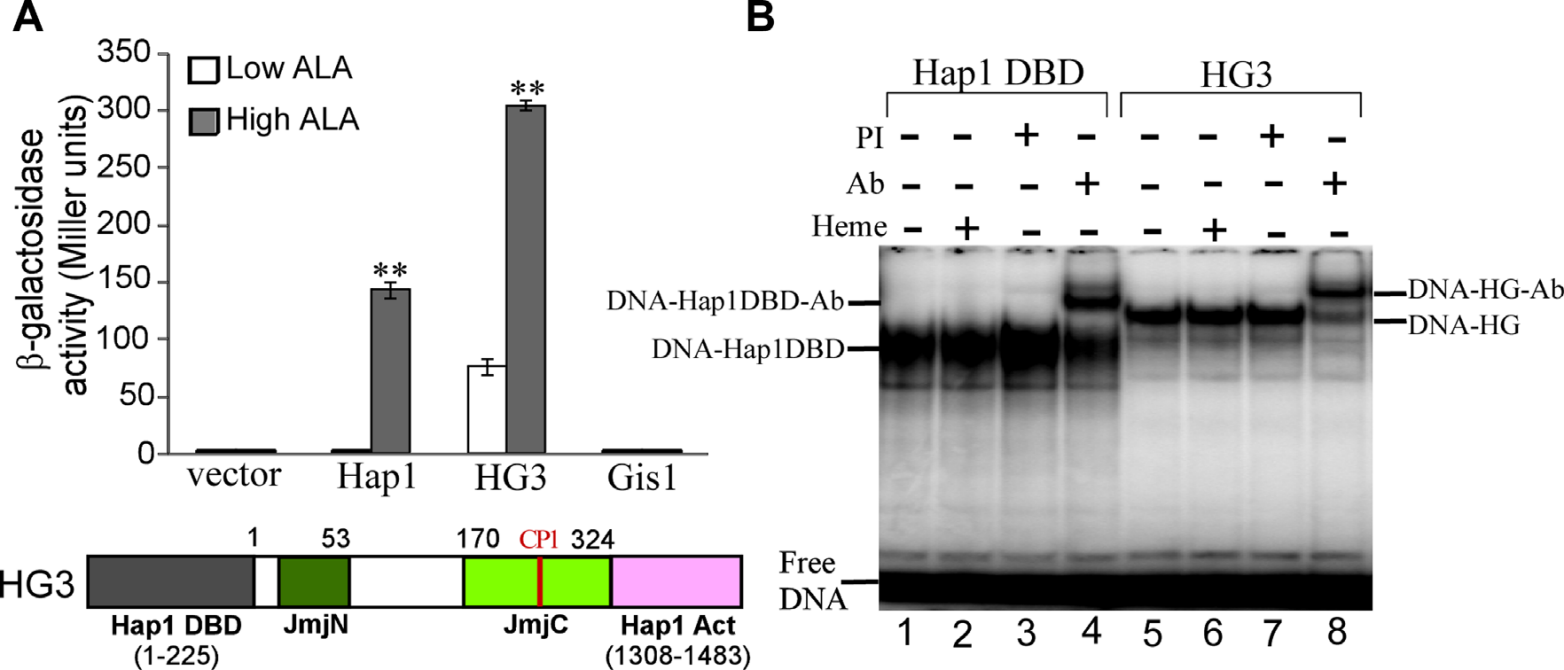
The JmjN + JmjC domain can confer heme regulation of transcriptional activity via an unrelated transcription factor. (A) Heme potentiates the transcriptional activity of Hap1-Gis1 fusion containing the Gis1 JmjN + JmjC domain. Yeast *Δgis1Δhem1* cells bearing the empty vector or the expression vector for wild type Hap1 or HG3 (containing Hap1 DNA-binding domain residues 1–225, Gis1 residues 1–324, and Hap1 residues 1308–1483), and the Hap1-binding UAS1-*CYC1-lacZ* reporter were grown in the presence of a low (2.5 μg/ml) or high (250 μg/ml) level of ALA. Then, cells were collected and β-galactosidase activities were measured. For statistical analysis, the levels in heme-deficient cells were compared to the levels in heme-sufficient cells with a Welch 2-sample t-test. **, *P* value <0.005. (B) Heme does not affect DNA binding by the fusion protein. EMSAs were performed with extracts prepared from cells expressing the Hap1 DNA-binding domain (DBD, lanes 1–4) or the fusion protein HG3 (lanes 5–8), as described in Materials and Methods. Anti-Hap1 antibodies were added in lanes 4 and 8, while PI was included in lanes 3 and 7.

To test whether heme binding to the JmjN + JmjC domain can be of functional relevance, we inserted the domain into a minimal transcription activator containing only the Hap1 DNA-binding and activation domains but lacking any heme regulatory regions (HG3, Figure 9A). As a control, we showed that the activity of full-length Hap1 is strongly activated by heme, as expected (Figure 9A) (58). Strikingly, while HG3 exhibited significant transcriptional activity in heme-deficient cells, like HG1 and HG2 (Figure 8A), its activity was further stimulated 4-fold in heme-sufficient cells. This result shows that the JmjN + JmjC domain can confer heme activation via an unrelated protein. Additionally, we showed that HG3 binds to DNA, as expected, and that its DNA-binding activity was not affected by heme (Figure 9B).

### Gis1 exhibits heme-activated H3K36 demethylase activity *in vitro*

**Figure 10.**
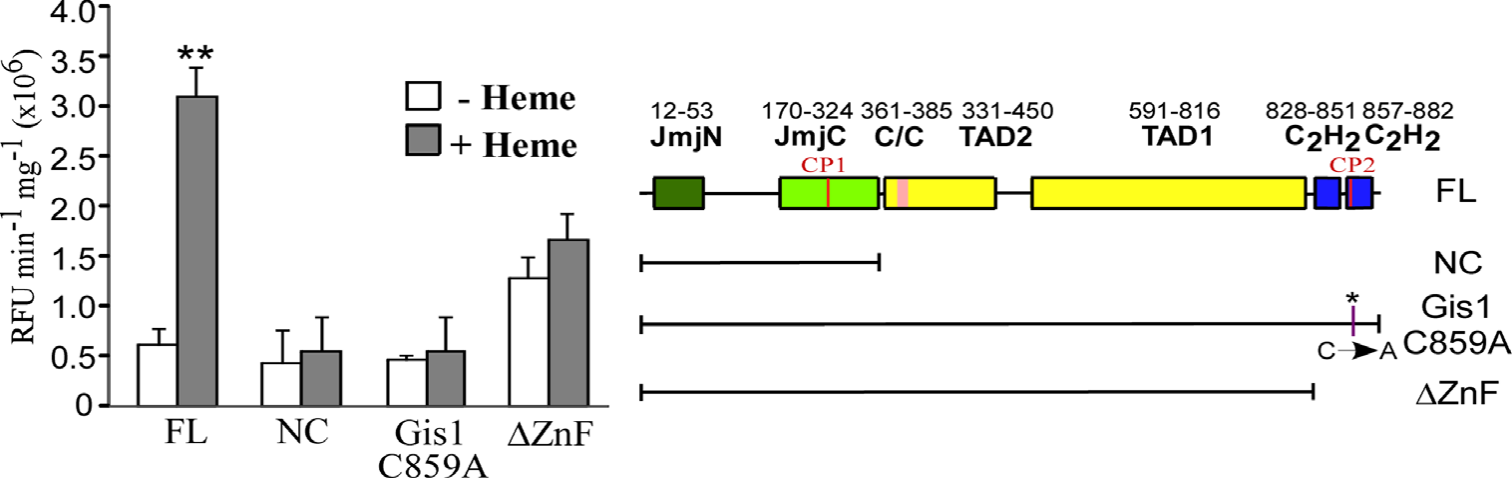
Gis1 demethylase activity is potentiated by heme. The H3K36me2 demethylase activities of purified full-length Gis1, NC, Gis1 C859A and ΔZnF (lacking Gis1 residues 828–894) proteins were measured as described in ‘Materials and Methods’ section. The assays were repeated multiple times. The data plotted here are averages from five different replicates. For statistical analysis, the activity in the presence of heme was compared to the activity in the absence of heme with a Welch two-sample *t*-test. **, *P*-value, 2 × 10^−5^.

Previous *in vivo* studies have suggested that Gis1 possesses H3K36 demethylase activity (20,32). To further ascertain if Gis1 has demethylase activity and if its activity is regulated by heme, we used a well-established, custom-made Epigenase Demethylase Activity/Inhibition Assay Kit (EpiGentek). We detected the activity of purified full-length Gis1 and NC containing only the JmjN + JmjC domain *in vitro*. We found that Gis1 and NC exhibit a similar level of demethylase activity on H3K36me2, in the absence of heme (Figure 10). In the presence of heme, the demethylase activity of full-length Gis1 was stimulated about 6-fold, while that of NC was not (Figure 10). Furthermore, when the Cys residue in CP2 is mutated to Ala, the C859A mutant’s histone demethylase activity was about the same in the absence or presence of heme (Figure 10). Notably, the ΔZnF mutant with the ZnF residues 828–894 deleted exhibited higher demethylase activity than full-length Gis1, suggesting that the ZnF masks the demethylase activity in the absence of heme. Heme activation of Gis1 demethylase activity of ΔZnF is largely abolished (Figure 10). These results show that the JmjN + JmjC domain indeed possesses H3K36me2 demethylase activity, but itself cannot mediate heme regulation of demethylase activity. The Gis1 C-terminal region containing the ZnF appears to mask demethylase activity in the absence of heme and promotes heme binding and activation when heme levels increase.

## DISCUSSION

### Gis1 is a versatile signal transducer

The *GIS1* gene was initially isolated as a multi-copy suppressor of a snf1/mig1/srb8 triple mutant (62). Gis1 was later found to be a key regulator promoting gene transcription after glucose depletion during and after diauxic shift, when cells shift their metabolism from glucose fermentation to oxidation of ethanol (24). During diauxic shift, the expression of over 2000 genes is affected (25). Gis1 has been shown to act both as a transcriptional activator and a repressor (31,45). Gis1 acts primarily on genes with PDS elements (30). These include many genes involved in stress response, phosphate metabolism, sulfur metabolism, amino acid metabolism, ribosome biogenesis and nutrient signaling (25–30). Gis1 and Msn2/4 are known to cooperatively mediate the entire Rim15-dependent transcriptional response during the diauxic shift (25). Through Rim15, Gis1 promotes nutrient signaling via both the TOR and PKA pathways (25,63). Additionally, it was suggested that Sch9 can directly act on Gis1 to promote its transcriptional activity (26). Thus, Gis1 is clearly a key signal transducer mediating signaling by major nutrient signaling pathways.

Furthermore, as a common downstream signaling transducer for Akt/PKB (Sch9), TOR and Ras signaling pathways, Gis1 plays important roles in stress resistance and life span extension in cells under calorie restriction and in long-lived mutants (25,27–29). As a yeast ortholog of the mammalian JMJD2/KDM4 protein family, Gis1 may have similar cellular functions as those of KDM4 proteins. For example, KDM4 associates with and stabilizes the DEP domain-containing mTOR-interacting protein (DEPTOR), a negative regulator of mTORC1/2 (64). Likewise, Gis1 and Tco89, a subunit of yeast TORC1 complex, have a negative genetic interaction (65). Such interactions provide links between modulators of KDM4/Gis1, such as the oncometabolite 2-hydroxyglutarate, with TOR signaling. Here, our data show that heme, a key metabolite in oxidative phosphorylation and mitochondrial respiration, directly regulates Gis1 activity. This function enables Gis1 to coordinate heme signaling with nutrient signaling. The dependence of Gis1 activity on heme fits well with the important function of Gis1 in promoting the transition of cells from glucose fermentation to ethanol oxidation during the diauxic shift.

### Structural environment impacts heme binding to Gis1 ZnF and JmjN + JmjC domains and heme regulation

C2H2 ZnF proteins likely constitute the largest family of regulatory proteins in mammals (59,66–68). They can interact with DNA, RNA and proteins, and modulate an array of biological processes, such as development, differentiation and tumor suppression (69,70). The ZnF enables Gis1 to bind to DNA, and is essential for transcriptional activation by Gis1. Remarkably, the ZnF conferred substantial heme regulation via an unrelated DNA binding domain (Figure 8A). Likewise, the JmjN + JmjC domain conferred substantial heme regulation in an unrelated transcription activator (Figure 9A). This is consistent with the data showing that heme binding to both NC and 750C causes protein conformational changes and decreases their protease sensitivity. Evidently, regardless of the functions of these protein domains in Gis1, their capability to bind heme and alter their conformations enables them to mediate heme regulation when protein architecture is properly constructed, as in the cases of HG2 and HG3 (Figures 8A and 9A). In many other cases, fusion or mutant proteins are often labile and inactive because protein architecture is not compatible with a stable protein domain structure and folding. For example, two fusion proteins (containing the Hap1 DNA-binding domain/full-length Gis1 and the Hap1 DNA-binding domain/Gis1 lacking the ZnF) and full-length Gis1 mutants with a mutated Cys residue in one of the CP motifs were labile and inactive (not shown). Previous data have shown that structural environment is crucial for heme binding by CP motifs and dictates whether heme binding causes any changes in protein functions (7,37–40). Note that strong heme-binding affinity of protein motifs does not generally correlate with high functional importance in heme signaling and regulation. In the case of Hap1, six clustered CP motifs with strong heme-binding affinity has no role in heme regulation while one CP motif is essential for heme regulation (35,39). Evidently, our data here also suggest that a module with weaker heme-binding affinity is required for heme regulation.

Intriguingly, while the Gis1 JmjN + JmjC domain binds to heme strongly (Figures 2 and 3) and can promote heme regulation via an unrelated protein (Figure 9), it does not promote heme activation of Gis1 activity (Figures 7A and 10). As such, one may ask what function heme binding to the JmjN + JmjC domain may serve. One intriguing possibility is that Gis1 may use the iron ion in heme, not a free iron ion, to catalyze H3K36 demethylation. Because Gis1 lacks the third residue (His) involved in iron binding with JmjC domain-containing proteins, as does its yeast paralog Rph1, it was previously suggested that Gis1 would not have demethylase activity (19). However, *in vivo* data from other labs (20,21,32) and our biochemical data shown here (Figure 10) strongly suggest that Gis1 possesses H3K36 demethylase activity. If the heme iron does indeed provide catalytic activity for demethylation by Gis1, this phenomenon may explain why Gis1 possesses demethylase activity even when it may not bind to iron. Further structural studies should uncover the detailed molecular events promoting the multiple fascinating functions of various Gis1 ZnF and JmjN + JmjC domains.

### Gis1 represents a novel class of multi-functional heme sensing and signaling regulators possessing both demethylase and transcriptional activities

Our biochemical and *in vivo* functional data provide strong experimental evidence showing that Gis1 possesses both transcriptional and demethylase activities. Further, we showed that heme promotes both transcriptional and demethylase activities of Gis1. While Gis1 transcriptional activity is very low in heme-deficient cells (Figure 1B), its demethylase activity is detectable in the absence of heme (Figure 9A). The addition of heme further potentiates demethylase activity. Intriguingly, while the ZnF binds to heme with a lower affinity than the JmjN + JmjC domain, the region encompassing the ZnF appears to be responsible for heme regulation of both demethylase and transcriptional activities. It is also worth noting that heme binding to the ZnF and JmjN + JmjC promotes oligomerization (Figure 5). It is conceivable that oligomerization may stimulate both demethylase and transcriptional activities of Gis1.

**Figure 11.**
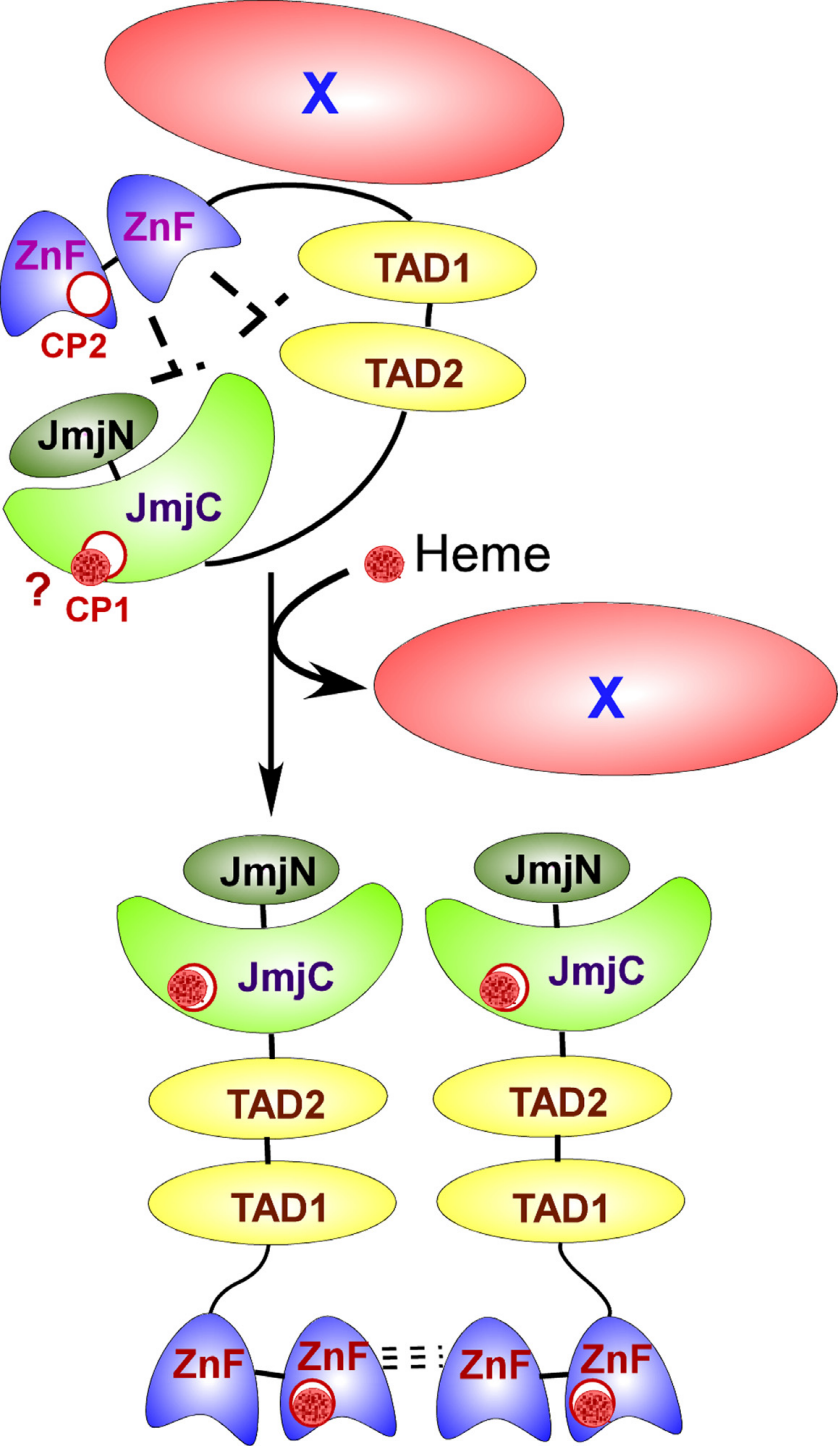
A cartoon illustrating how heme may regulate Gis1 demethylase and transcriptional activities. At lower heme levels, Gis1 transcriptional and demethylase activities are repressed by the region containing the ZnF and certain cellular proteins (designated as X). When Gis1 was purified from *Escherichia coli*, it was partially bound to heme at the high affinity site in the JmjN + JmjC domain, enabling Gis1 to exhibit partial demethylase activity. When heme level increases, heme can bind to the ZnF, causing Gis1 conformational changes and oligomerization, as well as the dissociation of X. Consequently, Gis1 demethylase and transcriptional activities are fully activated.

Based on our data, we propose the following tentative model for how heme acts to promote Gis1 activities (Figure 11): Under severely limiting heme conditions, Gis1 is free of heme and is inactive. Its activities are repressed by the ZnF region and likely other cellular proteins (X). The proposed repressive role of the ZnF is consistent with the data showing that deletion of the ZnF caused partial derepression of Gis1 demethylase activity (see Figure 10, compare the activities of full-length Gis1 and ΔZnF in the absence of heme). Many proteins have been shown to bind to Gis1 (71), and experiments in the authors’ lab have identified a series of Gis1-binding proteins (unpublished results). When heme levels become less limiting, heme likely binds to the JmjN + JmjC domain first because it binds to heme with high affinity. This binding would enable Gis1 to have some demethylase activity. As heme levels increase further, heme binds to the ZnF. Presumably, this will cause other cellular proteins to dissociate from Gis1 and Gis1 oligomerization, thereby leading to full activation of Gis1 transcriptional and demethylase activities. Although further experiments are necessary to validate this model, our data presented in this report clearly showed that Gis1 is a novel, multi-functional, heme-regulated complex signal transducer.

The mammalian orthologs of Gis1 include JMJD2A (KDM4A), JMJD2B (KDM4B) and JMJD2C (KDM4C) (72). They possess the JmjN and JmjC domains and PHD type ZnFs (Supplementary Figure S1). They have important functions in modulating histone methylation, hormonal signaling and oxygen signaling. They are implicated in many disease processes including tumorigenesis, cardiac hypertrophy and cardiomyopathy (3,5,6). These mammalian JmjC domain-containing proteins have at least one CP motif and many other residues capable of chelating heme. Thus, they all have the potential to sense heme and change their activity in response to heme. Additionally, both KDM4A and Gis1 are associated with TOR signaling proteins and likely play important roles in nutrient signaling (64,65). As such, Gis1 may exemplify this new class of heme regulatory histone demethylases and transcriptional regulators. Understanding molecular mechanisms governing Gis1-mediated signaling is likely to facilitate our understanding of mechanisms governing KDM4 protein functions.

## Supplementary Material

Supplementary Data
