# Supplementary material for: Heme promotes transcriptional and demethylase activities of Gis1, a member of the histone demethylase JMJD2/KDM4 family
Source: Nucleic Acids Res. 2017 Nov 6;46(1):215–28. doi: 10.1093/nar/gkx1051 (PMC5758875; doi:10.1093/nar/gkx1051)

SUPPLEMENTAL FIGURES

Figure S1. Sequence alignment of Gis1 with its mammalian orthologues JMJD2A/KDM4A, JMJD2B/KDM4B, and JMJD2C/KDM4C.

```
                                       JmjN
GIS1      ----MEIKPVEVIDGVPVFKPSMMEFANFQYFIDEITKFGI-ENGIVKVIPPKEWLELLE    55
JMJD2A    --MASESETLNPSARIMTFYPTMEEFRNFSRYIAYIESQGAHRAGLAKVVPPKEWKPRAS    58
JMJD2B    -MGSEDHGAQNPSCKIMTFRPTMEEFKDFNKYVAYIESQGAHRAGLAKIIPPKEWKPRQT    59
JMJD2C    MEVAEVESPLNPSCKIMTFRPSMEEFREFNKYLAYMESKGAHRAGLAKVIPPKEWKPRQC    60
                    :    : .* *:* ** :*. ::  : . *  . *:.*::*****

GIS1      GSPPAESLKTIQLDSPIQQQAKRWDKHENGVFSIENEYDNKSYNLTQWKNLAESLDSRIS    115
JMJD2A    ----YDDIDDLVIPAPIQQLVT----GQSGLFTQYN-IQKKAMTVREFRKIANS------    103
JMJD2B    ----YDDIDDVVIPAPIQQVVT----GQSGLFTQYN-IQKKAMTVGEYRRLANS------    104
JMJD2C    ----YDDIDNLLIPAPIQQMVT----GQSGLFTQYN-IQKKAMTVKEFRQLANS------    105
               :.:. : : :**** ..     :.*:*:  *  ::*: .: :::.:*:*

GIS1      QGDFNDKTLKENCRVDSQQDC-YDLAQLQILESDFWKTIAFSKPFYAVDENSSIFPYDLT    174
JMJD2A    -----------------DKYCTPRYSEFEELERKYWKNLTFNPPIYGADVNGTLYEKHVD    146
JMJD2B    -----------------EKYCTPRHQDFDDLERKYWKNLTFVSPIYGADISGSLYDDDVA    147
JMJD2C    -----------------GKYCTPRYLDYEDLERKYWKNLTFVAPIYGADINGSIYDEGVD    148
                            : *     : : ** .:**.::*  *:*..* ..:::   :

                                     JmjC
GIS1      LWNLNNLPDSINSSN------------RRLLTGQSKCIFPWHLDEQNKCSINYLHFGAPK    222
JMJD2A    EWNIGRLRTILDLVEKESGITIEGVNTPYLYFGMWKTSFAWHTEDMDLYSINYLHFGEPK    206
JMJD2B    QWNIGSLRTILDMVERECGTIIEGVNTPYLYFGMWKTTFAWHTEDMDLYSINYLHFGEPK    207
JMJD2C    EWNIARLNTVLDVVEEECGISIEGVNTPYLYFGMWKTTFAWHTEDMDLYSINYLHFGEPK    208
           **:  *   ::  :              *  *  *  * ** :: :  ******** **

GIS1      QWYSIPSANTDQFLKILSKEPSSNKENCPAFIRHQNIITSPDFLRKNNIKFNRVVQFQHE    282
JMJD2A    SWYSVPPEHGKRLERLAKGFFPGSAQSCEAFLRHKMTLISPLMLKKYGIPFDKVTQEAGE    266
JMJD2B    SWYAIPPEHGKRLERLAIGFFPGSSQGCDAFLRHKMTLISPIILKKYGIPFSRITQEAGE    267
JMJD2C    SWYAIPPEHGKRLERLAQGFFPSSSQGCDAFLRHKMTLISPSVLKKYGIPFDKITQEAGE    268
          .**::*  : .:: ::      .. :.* **:**:  : ** .*:* .* *.::.*   *

GIS1      FIITFPYCMYSGFNYGYNFGESIEFILDQQAVVRKQPLKCGCGNKKEERKSGPFSNLS--    340
JMJD2A    FMITFPYGYHAGFNHGFNCAESTNFATRRWIEYGKQAVLCSCRKDMVKISMDVFVRKFQP    326
JMJD2B    FMITFPYGYHAGFNHGFNCAESTNFATLRWIDYGKVATQCTCRKDMVKISMDVFVRILQP    327
JMJD2C    FMITFPYGYHAGFNHGFNCAESTNFATVRWIDYGKVAKLCTCRKDMVKISMDIFVRKFQP    328
          *:*****  ::***:*:* .** :*   :     *    * * :.  : . . * .

GIS1      --YDSNESEQRGSITDNDNDLF---QKVRSF--------DELLNHSSQELQNL--EDNKN    385
JMJD2A    ERYKLWKAGKDNTVIDHTLPTPEAAEFLKE----SELPPR-----AGNEEECPE-EDMEG    376
JMJD2B    ERYELWKQGKDLTVLDHTRPTALTSPELSSWSASRASLKAKLLRRSHRKRSQPKKPKPED    387
JMJD2C    DRYQLWKQGKDIYTIDHTKPTPASTPEVKAWLQRRRKVRK-----ASRSFQCAR-STSKR    382
            *.  :  :     *:          :                 : .. .       :

GIS1      PLFSNINMNR-----PQSSSLR----------------STTPNGVNQFLNMNQTTISRIS    424
JMJD2A    VEDGEEGDLKTSLAKHRIGTKRHRVCLEIPQEVSQSELFPKEDLSSEQYEMTECP-----    431
JMJD2B    PKFPGEGTAGAALLEEAGGSVKEEAGPEVDPEEEEEEPQPLPHGR--EAE-GAEE-----    439
JMJD2C    PKADEEEEVSDE--------------------VDGAE-VPNPDSVTDDLKVSEKS-----    416
                                                    .      :

GIS1      SPLLSRMMDLSNIVEPTLDDPGSKFK-RKVLTPQL--PQMNIPSNSSNFGTPSLTNTNSL    481
JMJD2A    --------AALAPVRPTHS--------SVRQVEDG----LTFPDYSDS------------    459
JMJD2B    --------DGRGKLRPTKAKSERKKKSFGLLPPQLPPPPAHFPSEEAL------------    479
JMJD2C    --------EAAVKLRNTEASSEEESSASRMQVEQNLSDHIKLSGNSC-------------    455
                       :. *                :       : . .
```

```
GIS1      LSNITATSTNPSTTTNGSQNHNNVNANGINTSAAASINNNISSTNNSANNSSSNNNVSTV     541
JMJD2A    -TEVKFEELKNVKLEEEDEE---------------------------EEQAAAALDLSVN     491
JMJD2B    ---WLPSPLEPPVLGPGPAA---------------------------MEESPLPAPLNVV     509
JMJD2C    ---LSTSVTEDIKTE--------------------------------DDKAYAYRSV---     477
                   :                                      :::     :

GIS1      PSSMMHSSTL-----NGTSGLG-G-----DNDDNMLALSLATLANSAT----ASPRLTLP     586
JMJD2A    PASVGGRLVFSGSKKKSSSSLGSGSSRDSISSDSE---TSEPLSCRAQG-QT---GVL--     542
JMJD2B    PPEVPSEE--LEAKPRPIIPMLYV-----VPRPGKAAFNQEHVSCQQAFEHFAQKGPTWK     562
JMJD2C    PS--------ISSEADDSIPLSSGYEKPEKSDPSELSWPKSPESCSSVAESN---GVL--     524
          *                   :            .         :

GIS1      PLSSPMNPNGHTSYNGNMMNNNSGNGSNGSNSYSNGVTTAAATTTSAPHN-----LSIVS     641
JMJD2A    -------TVHS----------Y----AKGDGRVTVGEPCTRKK-GSAARSFSERELAEVA     580
JMJD2B    EPVSPMELTGP----------EDGAASSGAGRME-----TKAR------------AGEGQ     595
JMJD2C    -------TEGE----------ESDVESHGNGLE-PGEIPAVPS--GERNSFKV-------     557
                                    : * .        :

GIS1      PNPTYSPNPLSLYLTNSKNPLNSGLAPLSPSTSN--IPFLKRNNVVTLNISREASKSPIS     699
JMJD2A    DEYMFSLEE--------NKKSKGRRQPLSKLPRHHPLVL--QECVS------DDETSEQL     624
JMJD2B    APSTFSKLK--------MEIKKSRRHPLGRPPTRSPLSVVKQEASS------DEEASP-F     640
JMJD2C    --PSIAEGE--------NKTSKSWRHPLSRPPARSPMTLVKQQAPS------DEELPEVL     601
               :            :  :.   **.    .  : .  ::         : .

GIS1      SFVNDYRSPLGVSNPL--MYSSTINDYSNGTGIRQNSNNINPLD-AGPSFSPLHKKPKIL     756
JMJD2A    TPEEEAEETEAWAKPLSQLWQNRPPNFEAEKEFNETMAQQAPHCAVCMIFQTYHQVE---     681
JMJD2B    SGEEDVSDPDALRPLLSLQWKNRAASFQAERKFNAAAARTEPYCAICTLFYPYCQAL---     697
JMJD2C    SIEEEVEETESWAKPLIHLWQTKSPNFAAEQEYNATVARMKPHCAICTLLMPYHKPD---     658
          :  ::  .  .    *   :..   .:      .    .  *       :    :

GIS1      NGNDNSNLDS--------------------NNFDYSFTGNKQESNPSILNNNTNNNDNY     795
JMJD2A    FGGFNQNC---G---NASDLAPQKQRTKPLIPEMCFTSTGCSTDINLSTP----YLEEDG     731
JMJD2B    QTEKEAPIASLGKGCPATLPSKSRQKTRPLIPEMCFTSGGENTEPLPANS----YIGDDG     753
JMJD2C    SSNEENDA---RWETKLDEVVTSEGKTKPLIPEMCFIYSEENIEYSPPNA----FLEEDG     711
              :                            :: :     . :             ::
                                              zinc finger PHD type1

                                              zinc finger C2H2 type
GIS1      RTSSMNNNGNNYQAHSSKFGENEVIMSDHGKIYICRECNRQFSSG---------HHLTRH     846
JMJD2A    TSILVSCKKCSVRVHASCYGVPPAKAS---EDWMCSRCSANALEEDCCLCSLRGGALQRA     788
JMJD2B    TSPLIACGKCCLQVHASCYGIRPELVN---EGWTCSRCAAHAWTAECCLCNLRGGALQMT     810
JMJD2C    TSLLISCAKCCVRVHASCYGIPSHEIC---DGWLCARCKRNAWTAECCLCNLRGGALKQT     768
           :  :       :.*:* :*         . : * .*  :               *
                 zinc finger PHD type1                 zinc finger C2HC type

            zinc finger C2H2 type                 zinc finger C2H2 type
GIS1      --KKS----------------------VHS-----GEKPHSCPRCGKRFKRRDHVLQHLN     877
JMJD2A    NDDRWVHVSCAVAILEARFVNIAERSPVDVSKIPLPRFKLKCIFCKKRRKRTAGCC----     844
JMJD2B    TDRRWIHVICAIAVPEARFLNVIERHPVDISAIPEQRWKLKCVYCRKRMKKVSGAC----     866
JMJD2C    KNNKWAHVMCAVAVPEVRFTNVPERTQIDVGRIPLQRLKLKCIFCRHRVKRVSGAC----     824
             :                       :.       .   .*  *  :*  *:
           zinc finger C2HC type                  zinc finger PHD type2
```

```
GIS1    KKIPCTQEMENTKLAES-------------------------------------------    894
JMJD2A  --VQCSHGRCPTAFHVSCAQAAGVMMQPDDWPFVVFITCFRHKIPNLERAKGALQSITAG    902
JMJD2B  --IQCSYEHCSTSFHVTCAHAAGVLMEPDDWPYVVSITCLKHKSGGH--AVQLLRAVSLG    922
JMJD2C  --IQCSYGRCPASFHVTCAHAAGVLMEPDDWPYVVNITCFRHKVNPNVKSKACEKVISVG    882
          : *:      : :  :
                 zinc finger PHD type2

GIS1    ------------------------------------------------------------    894
JMJD2A  QKVISKHKNGRFYQCEVVRLTTETFYEVNFDDGSFSDNLYPEDIVSQDCLQFGPPAEGEV    962
JMJD2B  QVVITKNRNGLYYRCRVIGAASQTCYEVNFDDGSYSDNLYPESITSRDCVQLGPPSEGEL    982
JMJD2C  QTVITKHRNTRYYSCRVMAVTSQTFYEVMFDDGSFSRDTFPEDIVSRDCLKLGPPAEGEV    942


GIS1    ------------------------------------------------------------    894
JMJD2A  VQVRWTDGQVYGAKFVASHPIQMYQVEFEDGSQLVVKRDDVYTLDEELPKRVKSRLSVAS    1022
JMJD2B  VELRWTDGNLYKAKFISSVTSHIYQVEFEDGSQLTVKRGDIFTLEEELPKRVRSRLSLST    1042
JMJD2C  VQVKWPDGKLYGAKYFGSNIAHMYQVEFEDGSQIAMKREDIYTLDEELPKRVKARFSTAS    1002

GIS1    --------------------------------------------------------894
JMJD2A  DMRFNEIFTEKE-VKQEKKRQRVINSRYREDYIEPALYRAIME-------------1064
JMJD2B  GAPQEPAFSGEEAKAAKR--PRVGTPLATEDSGRSQDYVAFVESLLQVQGRPGAPF1096
JMJD2C  DMRFEDTFYGADIIQGERKRQRVLSSRFKNEYVADPVYRTFLKSSFQKKCQKRQ--1056
```

Figure S2. Purified full-length Gis1 and Gis1 fragments N590, NC, 590C, 750C, and ZnF. Shown here are the electrophoresis patterns and the regions of full-length Gis1, and Gis1 fragments N590 containing residues 1-590, NC containing residues 1-324, 590C containing residues 591-894, 750C containing residues 751-894, and ZnF containing residues 828-894.

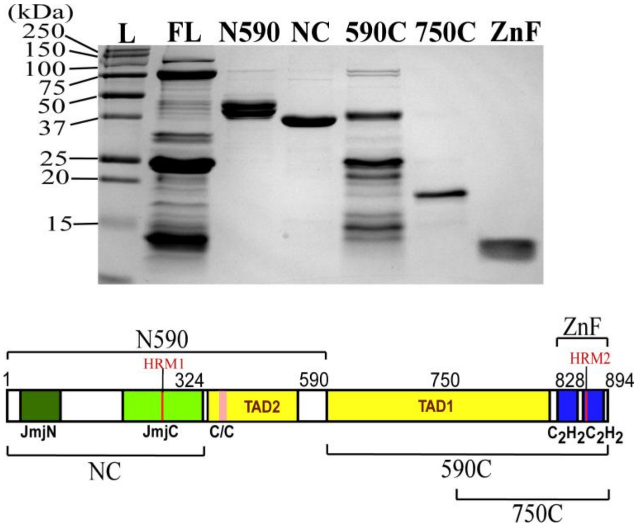

Figure S3. Absorption spectra of heme in the absence and presence of purified N590 (A) or 590C (B). line 1: 5 μM heme, line 2: 5 μM heme + 10 μM Gis1 protein, line 3: 5 μM heme + 10

mM imidazole, line 4: 5 μM heme + 10 μM Gis1 protein + 10 mM imidazole, line 5: 10 μM Gis1 protein.

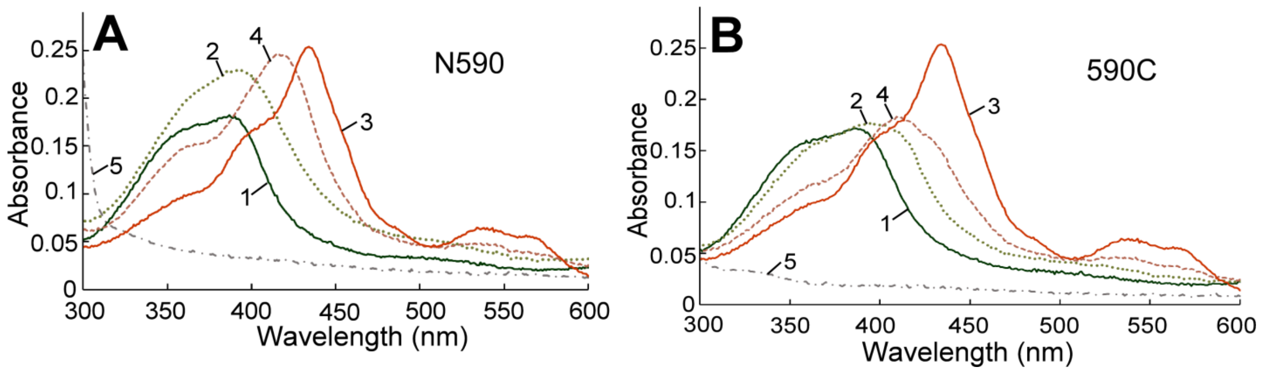

Figure S4. Absorption spectra of heme bound to Gis1 protein fragments in the presence of increasing concentrations of imidazole. (A) The shifting of heme-750C absorption peak from about 406 nm to 434 nm by increasing concentrations of imidazole. (B) The shifting of heme-ZnF absorption peak from about 406 nm to 434 nm by increasing concentrations of imidazole. (C) The shifting of heme-590C absorption peak from about 406 nm to 434 nm by increasing concentrations of imidazole. Line 1: 5 μM heme, line 2: 5 μM heme + 10 μM Gis1 protein, line 3: 5 μM heme + 10 mM imidazole, line 4: 5 μM heme + 10 μM Gis1 protein + 10 mM imidazole, line 5: 5 μM heme + 10 μM Gis1 protein + 20 mM imidazole, line 6: 5 μM heme + 10 μM Gis1 protein + 40 mM imidazole, line 7: 5 μM heme + 10 μM Gis1 protein + 80 mM imidazole, line 8: 5 μM heme + 10 μM Gis1 protein + 160 mM imidazole, line 9: 10 μM Gis1 protein.

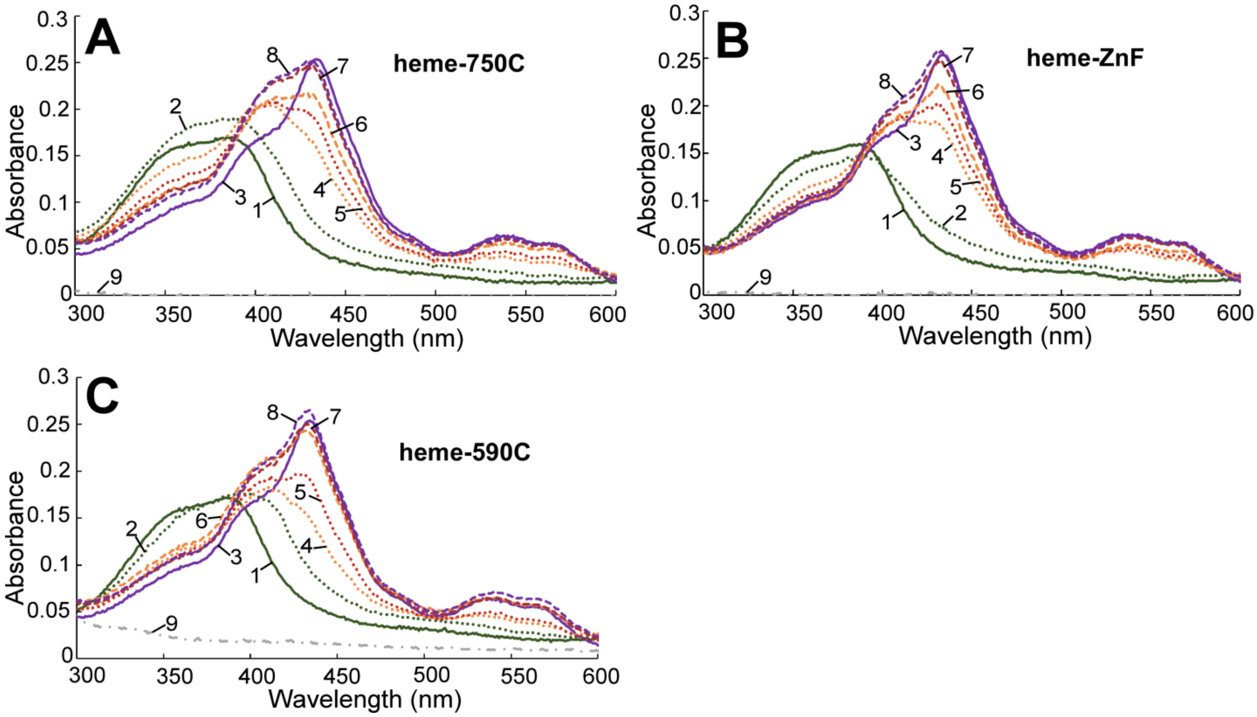

Figure S5. Heme does not affect elastase sensitivity of β-amylase. β-amylase in the absence or presence of heme was treated with increasing concentrations of elastase, as described in Materials and Methods. The presence of heme did not cause any difference in elastase sensitivity.

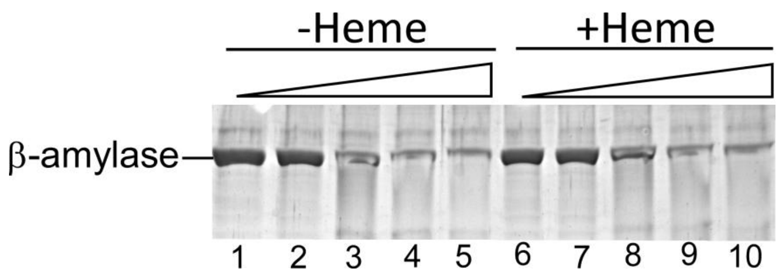

Figure S6. Heme does not affect Gis1 DNA binding by purified Gis1 (A) or Gis1 in extracts prepared from yeast cells expressing Gis1 (B). DNA-binding reactions were carried with purified

Gis1 or proteins extracted from cells expressing full-length Gis1 with a C-terminal FLAG tag in the presence of increasing concentrations of heme. An anti-FLAG antibody was included in lane 9 for super shift. Lane 1: free probe. (C) Heme does not affect significantly Gis1 binding to the *GRE1* promoter *in vivo*. ChIP-qPCR analysis of Gis1 binding to the GRE1 promoter in heme-deficient (low ALA) and heme-sufficient (high ALA) cells was carried out as described in Materials and Methods. Data from three independent experiments were calculated and plotted here. For statistical analysis, the levels in heme-deficient cells were compared to the levels in heme-sufficient cells with a Welch 2-sample t-test. **, p value <0.005.

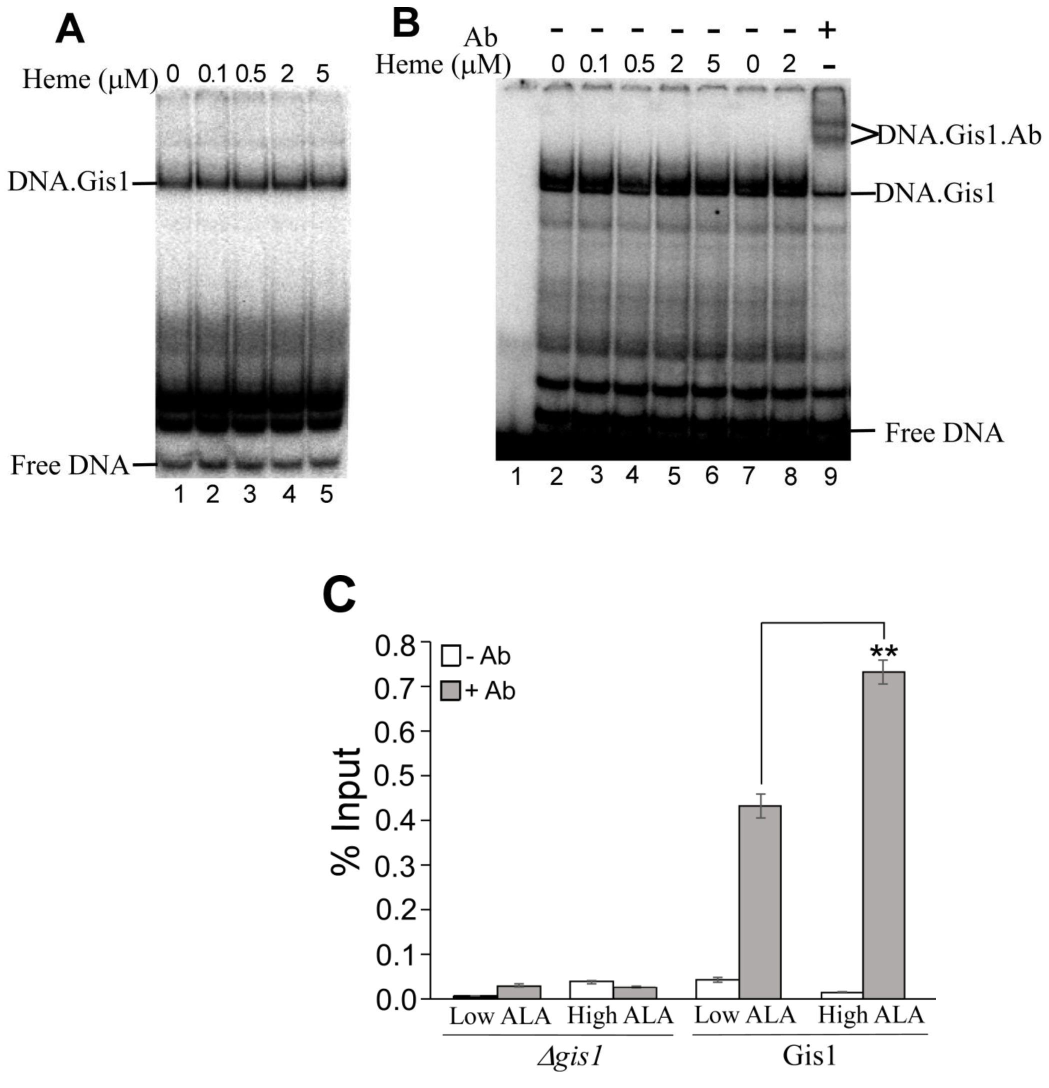

Figure S7. The nuclear localization of Gis1 and deletion proteins is not heme dependent. Yeast cells expressing FLAG-tagged full-length Gis1, ΔN/C, or ΔC proteins were grown under heme-deficient (L) or heme-sufficient (H) conditions. Cells were subjected to indirect immunofluorescence staining. The number of cells showing FLAG-tagged proteins in the nucleus (N) or cytosol (C) was counted and plotted here. The scale bar represents 1μm.

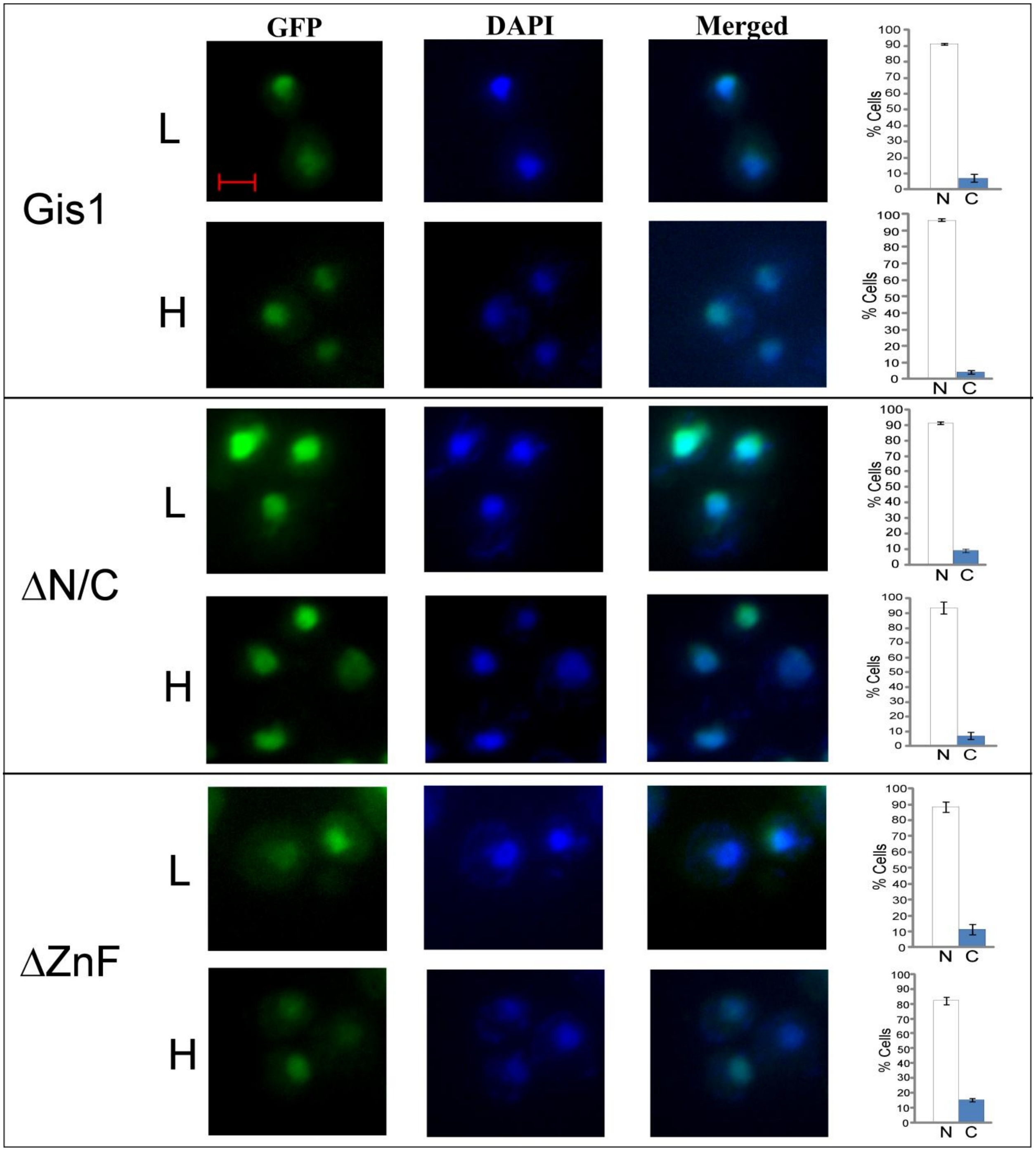

Supplement: Supplementary Data [file gkx1051_supp.pdf]
